# Synthesis, Activity, Toxicity, and In Silico Studies of New Antimycobacterial *N*-Alkyl Nitrobenzamides

**DOI:** 10.3390/ph17050608

**Published:** 2024-05-09

**Authors:** João P. Pais, Olha Antoniuk, David Pires, Tiago Delgado, Andreia Fortuna, Paulo J. Costa, Elsa Anes, Luis Constantino

**Affiliations:** 1 Research Institute for Medicines (iMed.UL), Av. Prof. Gama Pinto, 1649-003 Lisboa, Portugalt.delgado@edu.ulisboa.pt (T.D.); eanes@ff.ulisboa.pt (E.A.); 2 Faculdade de Fármácia, Universidade de Lisboa, Av. Prof. Gama Pinto, 1649-003 Lisboa, Portugal; 3 Centro de Investigação Interdisciplinar em Saúde (CIIS), Faculdade de Medicina, Universidade Católica Portuguesa, Estrada Octávio Pato, 2635-631 Rio de Mouro, Portugal; 4 Instituto de Biosistemas e Ciências Integrativas (BioISI) and Departamento de Química e Bioquímica, Faculdade de Ciências, Universidade de Lisboa, 1749-016 Lisboa, Portugal; pjcosta@ciencias.ulisboa.pt

**Keywords:** tuberculosis, nitrobenzamides, DprE1, mycobacteria

## Abstract

Tuberculosis (TB) is a disease that plagues the frailest members of society. We have developed a family of *N*-alkyl nitrobenzamides that exhibit promising antitubercular activities and can be considered a structural simplification of known inhibitors of decaprenylphosphoryl-β-D-ribofuranose 2′-oxidase (DprE1), an essential *Mycobacterium tuberculosis* (Mtb) enzyme and an emergent antitubercular target. Hereby, we report the development of these compounds via a simple synthetic methodology as well as their stability, cytotoxicity, and antitubercular activity. Studying their *in vitro* activity revealed that the 3,5-dinitro and the 3-nitro-5-trifluoromethyl derivatives were the most active, and within these, the derivatives with intermediate lipophilicities presented the best activities (MIC of 16 ng/mL). Additionally, in an *ex vivo* macrophage model of infection, the derivatives with chain lengths of six and twelve carbon atoms presented the best results, exhibiting activity profiles comparable to isoniazid. Although the proof is not definite, the assessment of susceptibility over multiple mycobacterial species, together with the structure similarities with known inhibitors of this enzyme, support DprE1 as a likely target of action for the compounds. This idea is also reinforced by the docking studies, where the fit of our more active compounds to the DprE1 binding pocket is very similar to what was observed for known inhibitors like DNB1.

## 1. Introduction

Tuberculosis is a respiratory tract disease caused by the *Mycobacterium tuberculosis* species complex, which is spread between people through aerosols containing as little as one to three bacilli [1]. Although TB mostly affects the lungs, it may adversely impact the brain, kidneys, or spine. Despite being a treatable disease, tuberculosis still remains one of the leading causes of death globally, being the second most common single infectious agent-related cause of death (ranking above HIV/AIDS and only second to COVID-19 in 2022) [2,3,4]. The COVID-19 pandemic has reversed years of global progress in tackling tuberculosis [5] and, for the first time in over a decade, TB deaths have increased, and only now are starting to return to pre-pandemic levels, according to the World Health Organization’s 2023 Global TB report [4].

**Figure 1:**
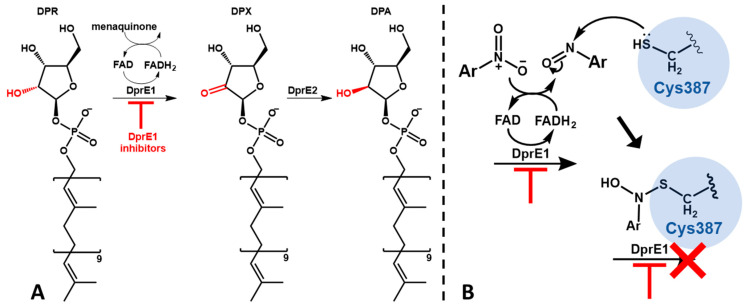
Schematic representation of the DprE1–DprE2 complex function and its inhibition; (**A**)—epimerization of DPR to DPA; (**B**)—mechanism of activation and DprE1 inhibition of nitroaromatic suicide inhibitors.

Most cases of tuberculosis can be treated and the spread of infection can be prevented with prompt diagnosis and six months of first-line antibiotic treatment [4]. However the increasing appearance of multidrug-resistant (MDR) and extensive drug-resistant (XDR) TB makes clear the need for new drugs that can be effective and used in shorter therapeutic regimens [6]. Numerous novel therapeutic targets have been established in recent years with the effort to develop new effective anti-TB therapeutics, many of which target essential proteins implicated in the synthesis of the cell wall components [7,8]. One relevant example is DprE1, considered one of the promising targets for the development of new anti-TB drugs [9]. DprE1 is a component of the DprE1–DprE2 complex, an heterodimeric protein that catalyzes the epimerization of decaprenylphosphoryl-D-ribose (DPR) to decaprenylphosphoryl-D-arabinose (DPA) (Figure 1A) [10]. DPA is a vital precursor in the production of both lipoarabinomannan and arabinogalactan, and is essential in cell wall biosynthesis [11,12]. One of the reasons that makes DprE1 so promising is its intracellular location; since it is located in the periplasmatic space of the mycobacteria, the only barrier to the action of its inhibitors is the cell wall [13].

Inhibitors of DprE1 are generally subdivided into covalent and non-covalent inhibitors. The mechanism of covalent inhibition is based on the formation of a non-reversible covalent bond between the inhibitor and the cysteine 387 residue (Cys387) of DprE1. For this to happen, covalent inhibitors make use of an aromatic nitro moiety that, upon interaction with the active site of DprE1, undergoes a reduction of the nitro group to nitroso, which then forms a covalent adduct with Cys387 irreversibly hindering the protein’s function (Figure 1B) [14,15]. This residue is profoundly conserved in mycobacteria, aside from in *Mycobacterium avium* and *Mycobacterium aureum*, where cysteine is replaced by alanine and serine, respectively [16,17]. 

Our research group has studied benzoic acid and its derivatives for their antimycobacterial potential, specifically against the Mtb H37Rv strain, attempting to develop alkyl esters as prodrugs capable of increasing the weak acids’ antitubercular activity [18,19]. We provided proof that mycobacteria may quickly hydrolyze a range of organic acid esters [20,21] and that esters demonstrate greater *in vitro* activity than that of the corresponding free organic acids [22,23], indicating that they are appropriate prodrugs for the substances, helping the molecules enter the cells and releasing the free acid.

In previous work, we found that alkyl esters of 3,5-dinitrobenzoic acid showed very relevant antitubercular activities [24]. The activity was much higher than anticipated by Zhang et al. [18] based on the pka of the liberated free acid, indicating that the presence of the nitro groups might be important for the mechanism of action. Our inability to correlate the activity with ester hydrolysis rates led us to hypothesize that these compounds might act directly as active drugs rather than prodrugs. If the compounds could be acting as drugs, then a series of isosteres could be explored for activity.

Building on this, after exploring the potential of nitrobenzoates and nitrothiobenzoates [25], we decided to explore the antimycobacterial potential of bioisosteric amide analogues. Only two 3,5-dinitrobenzamides with linear alkyl chains were reported in the literature as having antimycobacterial activity [26], but they were somehow disregarded, as their activity was lower when compared with other more complex derivatives containing a terminal aromatic moiety (such as in DNB1 or DNB2). The reason for that is probably because the authors unfortunately only explored the derivatives with C6 and C16 *N*-alkyl groups. Because we knew from our previous work with nitro-containing esters that the alkyl chain could be optimized for activity by modifying the chain length of the compounds [25], we decided to apply the same approach to the *N*-alkyl benzamide analogues with the objective of improving their antimycobacterial efficacy.

Our initial efforts focused on synthesizing a series of nitrobenzamides with *N*-alkyl chains ranging from four to sixteen carbon atoms and evaluating their antitubercular activities. Encouraged by the results obtained on the activity of these compounds, we extended our study to include their performance in a macrophage infection model. Given the fears associated with nitroaromatic compounds in drug development [27], the cytotoxicity of the compounds reported upon in this study was assessed using human macrophages. We also decided to study the stability of the compounds in buffer, plasma and mycobacterial homogenate. Buffer stability studies were used to evaluate the chemical stability of the compounds at a pH level of 7.4 and plasma stability studies served to evaluate if the compounds exhibit stability, which allows for the determination of activity *in vivo*. The stability of the compounds in the mycobacterial homogenate was examined to determine the effect of mycobacterial enzymes on the compounds. Since we had no access to purified DprE1 to directly test the inhibition of purified DprE1, we evaluated the effects of our compounds on various mycobacteria species known to have differing responses to DprE1 inhibitors. In parallel, computational docking studies were performed to analyze how our compounds fit within the DprE1 binding pocket, compared to DNB1 and DNB2, which are known inhibitors of DprE1. These studies supported the potential of DprE1 as a possible target for our compounds, and lead to the discovery of new DNBs with antitubercular activities comparable to established benchmarks like DNB1. 

## 2. Results

### 2.1. Compound Library and Antitubercular Activity

A library comprising four series of benzamides, with 4-NO_2_, 3,5-NO_2_, 3-NO_2_-5-CF_3_, and H- substitution in the aromatic ring and *N*-alkyl groups of varying lengths, was obtained following a standard synthetic approach. The objective was to explore the effect of nitro substitution on the activity of the compounds and at the same time, study the effect of the *N*-alkyl group length on the activity of each series. The compounds obtained, as well as their corresponding antitubercular activity, determined in terms of minimal inhibitory concentration (MIC) and minimal bactericidal concentration (MBC), are presented in Table 1.

The first observation is that the compounds with a nitro substituent in the 3-position of the aromatic ring presented activities that were significantly higher than the other compounds. The 3,5-dinitro and 3-NO_2_-5-CF_3_ series presented activities that were significantly higher than the other two series; in addition, the most active compounds in the series containing a 3-nitro substitution (**11**, **12**, and **18**) presented MIC ca 2000 times lower than the most active compounds in each of the other two series (**3** and **6**).

**Figure 2:**
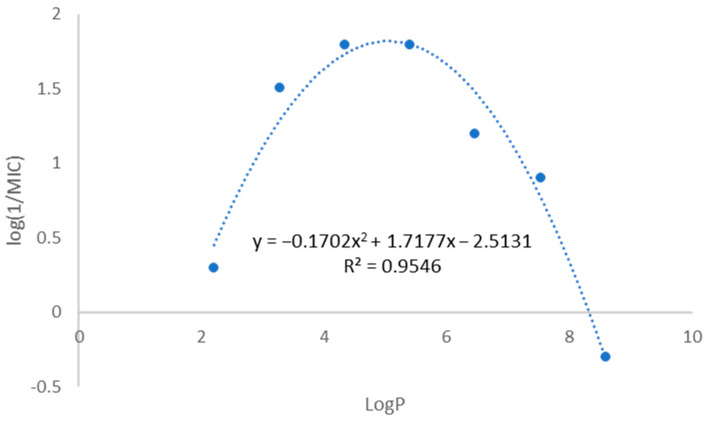
Graphical representation of the correlation between log*P* and log(1/MIC) values for the *N*-alkyl-3,5-dinitrobenzamide series (compounds **9** to **15**).

The library contains compounds with very different lipophilicities; as predicted, the octanol–water partition coefficient (log*P*) ranged from 2.21 to 8.59. When we analyzed the library as a whole, no correlation between the log*P* value and the antitubercular activity was found. However, when we look at each series independently, a different pattern emerges; in all the series, the more active compounds are the ones with intermediate lipophilicity (and number of carbons in the alkyl chain between six and ten). Figure 2 represents the correlation between log*P* and MIC values for the 3,5-dinitro-substituted benzamides. A polynomial correlation between the log*P* value and the antimycobacterial activity can be identified, indicating the possibility that this is indeed a main parameter modulating activity.

### 2.2. Susceptibility Assessment of Multiple Mycobacterial Species

The difference in activity found between the series of compounds tested could be indicative that the two more active series are most likely acting by a diverse mode of action to the remainder of the compounds. Our *N*-alkyl-3,5-dinitrobenzamides can be considered a simplification of the classic antitubercular amide DNB1 [29], containing a simple lipophilic alkyl group instead of a more complex substituent in the amide nitrogen. Similarly, the *N*-alkyl-3-nitro-5-trifluoromethylbenzamides are in turn a simplification of the active derivative A1 (itself a bioisostere of DNB1) [30] or a simplification of CT319 [31]. Both A1 and CT319 are active 3-nitro-5-trifluoromethylbenzamide derivatives that contain a trifluoromethyl group in the 5-position instead of a nitro group. Those three compounds act on DprE1 [29,30,31]. Although not definite, a simple approach to test if DprE1 could be a possible target for these compounds consists in challenging a range of bacterial strains with our compounds and known DprE1 inhibitors [16,32]. Since the different bacterial strains possess diverse sensitivities to DprE1 inhibition, a bioactivity profile of action could be established. Hence, the susceptibility of *M. tuberculosis*, *M. bovis BCG*, *M. avium*, and *M. smegmatis* was assessed and compared to DNB1 and DNB2, which are known DprE1 inhibitors [29,33]. The results are presented in Table 2. Compounds from a previous study [25] were also tested together with the amides, which are the compounds O9–O15, comprising the ester analogues of compounds **9**–**15**.

The first part of Table 2 shows the bioactivity profile of DprE1 inhibitors when compared with two control drugs, isoniazid (INH) and para-aminosalicylic acid (PAS). INH is highly active against Mtb and *M. bovis*, and moderately active against *M. smegmatis* and *M. avium*. PAS only presented high activity against Mtb and *M. bovis.* The known DprE1 inhibitors DNB1 and DNB2 possessed high activity against Mtb and *M. bovis*, moderate activity against *M. smegmatis* (MIC of 0.5 µg/mL), and a lower activity against *M. avium* (MIC of 32 and 64, respectively). The results obtained with the DprE1 inhibitors are in accordance with the literature. Indeed, *M. avium* does not have the codon that codes for Cys387 in DprE1, with cysteine being replaced by alanine [16]. This mutation confers natural resistance to the DprE1 inhibitors. The NfnB nitroreductase is overexpressed in *M. smegmatis*, which may cause the drug to become inactive due to the reduction of the nitro group to an amino group [17,34]. The other two species, *M. bovis* BCG and *M. tuberculosis* H37Rv, are equally susceptible to DprE1 inhibitors [35].

The second part of Table 2 shows the activities of compounds **3**–**15** against the same mycobacteria. Comparing the profile of DprE1 inhibitors DNB1 and DNB2 with the most active 3,5 dinitro-substituted derivatives (compounds **11** and **12**), we can observe similar effects: while the MIC values for *M. bovis* are the same or slightly increased when compared to the Mtb values, the susceptibility of *M. smegmatis* was decreased and *M. avium* was shown to be completely resistant. Furthermore, this profile is not observed for amides 3 and 7, which present different structures. Compound **3** does not present a nitro substituent and compound **7** presents a single nitro substituent, but it is in the 4-position of the aromatic ring.

Finally, in the present study we also determined the activity of esters O9–O15, which are structurally similar to the class of more active amides found in our studies (**9**–**15**). All compounds whose identification starts with an O are ester isosteres of the corresponding *N*-alkyl-3,5-dinitrobenzamides with the same number. Although the activity of the esters is lower than the corresponding amides, two different patterns can also be observed. The more active esters (O11 and O12) present a similar activity for Mtb and *M. bovis,* no significantly different activity for *M. smegmatis*, and a complete loss of activity against *M. avium*. The less active esters (compounds O9 and O15) do not show the same marked differences between the different mycobacteria species studied.

### 2.3. Cytotoxicity Evaluation in Human Macrophages

Given that aromatic compounds containing nitro groups are associated with adverse effects, namely due to their toxicity, genotoxicity, mutagenicity, and/or carcinogenicity [36,37], the cytotoxicity of the compounds under study was assessed on the THP-1 human monocytic cell line (ATCC56 TIB202). Although there are no guidelines for admissible values, cytotoxicity can be estimated by determining the lethal concentration for 50% of the cell population, LC_50_ [38]. The lower the LC_50_ value, the lower the lethal concentration of the compound used; hence, there is a higher toxicity to eukaryotic cells. Furthermore, the ratio between the LC_50_ and MIC values (LC_50_/MIC) was determined in order to better sort the compounds according to their toxicity [38]. This ratio also serves as an indicator of a compound’s selectivity between mycobacterial and eukaryotic cells, with a higher LC_50_/MIC ratio suggesting greater potential interest in the compound. The results obtained are presented in Table 3.

The amides under study have LC_50_ values between 14 and 360 μg/mL. However, since the LC_50_ values obtained for such compounds correspond to concentrations much higher than their MIC values, this results in high values of LC_50_/MIC, or good selectivity to the mycobacterial targets. For example, for compound **11**, LC_50_/MIC = 6063, which means that the LC_50_ value of this compound is 6063 times higher than its MIC value. Hence, the results show that there is a large difference between the concentration of the compound that impacts the mycobacteria (MIC) and the one that impacts human cells (LC_50_).

### 2.4. Biological Activity in Macrophage Model of Infection

**Figure 3:**
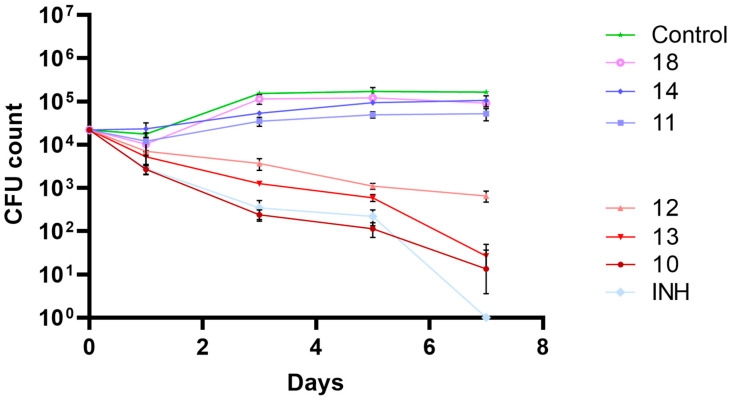
Biological activity of the most active amides under study in a macrophage model of infection. The results presented are the average of triplicate experiments and the error bars report a standard deviation (σ). Compounds and isoniazid (INH) were tested at 0.15 μg/mL.

Since the compounds under study showed promising antitubercular activities, their activity in an infection model was studied, namely by evaluating their antimycobacterial activity in THP-1 human macrophages infected with *M. tuberculosis* H37Rv as a model of mycobacterial infection. To assess the mycobacterial intracellular survival/killing, bacteria that survive the treatment were recovered from infected treated macrophages and plated on solid medium. CFU counts were assessed on days 0, 1, 3, 5, and 7 post infection. The results obtained are presented in Figure 3. 

Figure 3 shows the effects of the compounds on the intracellular killing in infected macrophages relative to the control (non-treated culture). INH was used as a positive control. At the dose tested (0.15 μg/mL), not all compounds that showed good activity *in vitro* confirmed the activity in the macrophage assay. The compounds that presented higher activity both *in vitro* and *ex vivo* in infected macrophages were compounds **10**, **12**, and **13**. Compound **18**, the most active compound in the 3-nitro-5-trifluoromethyl benzamide series, did not show good activity in macrophages. The same can be said about compound **11**, which was one of compounds that showed higher activity *in vitro*. The compound that had the best results in both assays was compound **12**.

### 2.5. Stability Studies

In previous studies, the compounds under study were esters and an attempt at establishing a correlation between antimicrobial activity and their stability in multiple biological media was made. However, since the esters were prone to hydrolysis, it was difficult to state that the compounds were not acting as prodrugs of weak acids, which are known for light antimycobacterial activity [18,39]. The amide analogues that are discussed here emerged as analogues with potentially higher resistance to hydrolysis; hence, their stability was also assessed. 

For this assessment, phosphate-buffered saline (PBS), human plasma, and *M. smegmatis* homogenate were used and all the compounds were incubated with the media. The detection of the respective free acid allowed the determination of the pseudo first order rate of the hydrolysis (for the compounds that decomposed faster) and the percentage of decomposition at 72 h (for the most stable compounds). The results are presented in Table 4. No other products other than the free acid resulting from hydrolysis were detected in the incubations. The stability of the compounds was initially tested with PBS at a pH level of 7.4 and no degradation was observed over two weeks, indicating that all the degradation observed in human plasma and mycobacterial homogenate is mainly enzymatic.

These results show that the amides are highly resistant to hydrolysis in the media assayed, with only the shorter-chained derivatives being hydrolyzed. Such an effect is in accordance with what was observed for the corresponding esters [25], where the shorter-chained derivatives showed the most hydrolysis. Nevertheless, short-chain *N*-alkyl amides were more easily hydrolyzed in mycobacterial homogenate than in plasma in our incubation conditions. Since the mycobacterial homogenate is richer in enzymes than the plasma due to being made from the whole mycobacterial cell [20], it is probably richer in amidases and thus can more easily perform the hydrolysis of short-chain *N*-alkylamides. Our more active compounds were not easily hydrolyzed by the mycobacterial homogenate.

### 2.6. Computational Studies

Considering the structure of our nitro-containing compounds, the difference in activity observed, the structural similarity with known DprE1 inhibitors, and the results obtained regarding the activity against the different mycobacterial species presented, DprE1 is the most attractive target for the compounds presented in this study. With the aim of further exploring this possibility, a docking-based computational study was performed, where the position of our compounds within the binding pocket of DprE1 was studied and compared with that of DNB1 and DNB2. The 4P8L PDB entry was chosen for this purpose since it is the only structure with a completely resolved DprE1 enzyme. In this study, five docking runs were performed using three scoring functions (AutoDock, Vina, and Vinardo), and their top scoring pose was selected. Next, the poses obtained were analyzed in relation to their distance to the mechanistically relevant locations for DprE1 inhibition, the cysteine 387 residue and the FAD cofactor. Using these poses, the distance of the nitro groups to both cysteine 387 (ND_Cys387_) and FAD (ND_FAD_) was calculated and since some compounds do not contain a nitro group, the center of the aromatic moiety was also considered (CD_Cys387_ and CD_FAD_). This analysis allows us to study the fit of the compounds in the binding pocket in a way that is less dependent on arbitrary interactions and more relevant to the expected mechanism of inhibition.

**Figure 4:**
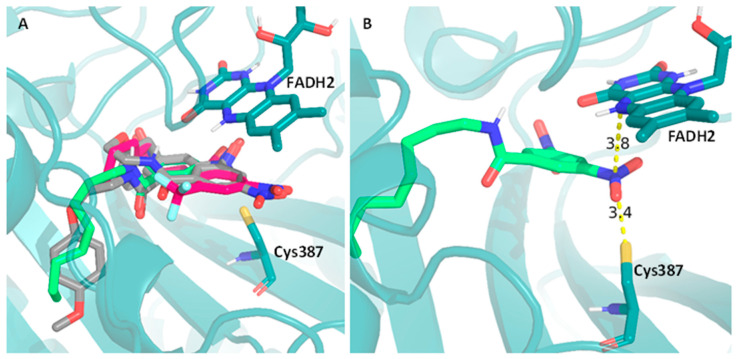
Representation of the best binding poses obtained from the docking computational study, between a PDB crystal structure (PDB: 4P8L) and (**A**)—the compounds DNB1 (gray), **11** (green), and **18** (pink); (**B**)—distance between compound **11** (green) and both the cysteine residue (Cys387) and the FAD cofactor (FADH_2_). Compounds **11** and **18** were selected for the figure as illustrative examples of the respective families.

To exemplify this analysis, we selected compound DNB1 (gray), compound **11** (green), and compound **18** (pink), and the overlap of their best docking poses is presented in Figure 4A. These compounds were selected as representatives of their respective families, the 3,5-dinitro-substituted and 3-nitro-5-(trifluoromethyl)-substituted families, respectively, since all compounds within these families have similar distances to both the cysteine residue and the FAD cofactor. Since, according to the mechanism of action of DNBs (Figure 1B), the nitro group must firstly be activated by the FADH_2_ and then quickly attacked by the sulfur atom of the cysteine residue, these moieties can the considered a pre-reactive triad (Figure 4B). As Figure 4A shows, all the selected compounds are in the proper position to form this triad.

The results that led to the “best fit” of the compounds to the binding pocket, which correspond to the poses with shorter ND_Cys387_, ND_FAD_, CD_Cys387_, and/or CD_FAD_ distances, are presented in Table 5. 

The sum of the distances to cysteine 387 and FAD was included (both ΣNDist and ΣCDist) as an easier way to retrieve some conclusions regarding their spatial position in the binding pocket of DprE1. Firstly, we can observe that, in general, within the same family of compounds, the distances measured are similar, indicating that the variation in the alkyl chain length does not significantly impact the fit of the compounds to the binding pocket, even though their score values indicate a tendency to increase with the increasing chain length. Furthermore, we can see that both ΣNDist and ΣCDist can be used to distinguish the most active families of compounds from the least active families. For example, compounds **5**–**8**, which have higher MIC values (512–32 µg/mL), have higher ΣNDist (25.110–8.941 Å) and ΣCDist (~24 Å) than the family of compounds **9**–**15** that are considerably more active (MIC values of 2–0.016 µg/mL), for which these distances were notably lower (ΣNDist ~7 and ΣCDist ~10 Å). Moreover, the most active family of compounds has similar values of ΣNDist and ΣCDist when compared to DNB1 and DNB2. 

## 3. Discussion

The benzamides presented here were easily obtained by reacting the acid chloride with the corresponding amide. Detailed synthesis procedure yields and results which are not presented in the manuscript are available in the Supplementary Materials.

The large difference in activity observed between compounds **9**–**20** versus others suggests distinct mechanisms of action. The *in vitro* activity of these benzamides is primarily centered on the presence of a nitro group at the 3-position of the benzene ring. The substitution of this nitro group or its relocation to the 4-position significantly diminishes activity, echoing findings by Christophe et al. [29].

The fact that most of the activity was maintained by the substitution of the nitro group in the 5-position of the aromatic ring by a trifluoromethyl group is also in accordance with the literature [30,40]. In an interesting study, Li et al. developed nitrobenzamide compounds by simplifying the structure of PBTZ169 [30]. They found that substituting the nitro group with a CF3 group slightly reduced the compounds’ activity. However, the overall activity remained comparable to the original nitro derivatives, suggesting that a nitro group could be replaced with a CF3 group to address pharmacokinetic or toxicological concerns.

Lipophilicity is also important, but it only modulates the activity of each series with *N*-alkyl chain lengths of six to ten carbons being optimal. Since our computational studies indicate that the lengths of the alkyl chain only interfere slightly with the position of the inhibitor in the binding pocket of DprE1, it seems that the optimal lipophilicity of the compounds is mainly needed to allow the compounds to access the periplasmatic space where DprE1 is located. 

The relationship between the log*P* value and antimycobacterial activity in the 3,5-dinitro-substituted derivatives indicates lipophilicity as a critical factor (Figure 2). Considering the lipid-rich composition of the mycobacterial cell wall, increasing lipophilicity may enhance membrane permeation; however, it also reduces water solubility, which poses challenges in biological assays, especially for compounds with a *N*-alkyl chain longer than ten carbons. Therefore, it is conceivable that longer alkyl chains have the capacity to exhibit comparable or even higher intrinsic DprE1 inhibition, but their solubility prevents its expression, leading to the parabolic behavior observed in Figure 2.

To support our hypothesis that our compounds inhibit DprE1, we evaluated their effectiveness against *M. tuberculosis*, *M. bovis* BCG, and *M. avium* and compared these results with those of known DprE1 inhibitors, DNB1 and DNB2. Taking into consideration the limitation that *M. avium* is naturally resistant to most antibiotics [23], we can indeed observe that the amides **11** and **12** showed high activity in *M. bovis* and *M. tuberculosis*, which are naturally sensitive to DprE1 inhibitors; however, the activity is completely lost in *M. avium*, which has a mutation in the DprE1 enzyme, namely an alanine residue in the place of Cys387. This cysteine is fundamental for the mode of action of covalent DprE1 inhibitors as it is its thiol group that reacts with the nitroso group of the activated inhibitor (Figure 1). Similar results were presented by the benzothiazinone BTZ043, which was tested by Caroline et al. [16], where BTZ043 showed high activity in *M. tuberculosis*, which was lost in *M. avium*. The NfnB nitroreductase is overexpressed in *M. smegmatis*, which may cause the drug to become inactive due to the reduction of a nitro group to amine, which is unable to react with the cysteine residue. The fact that our more active 3,5-dinitrobenzamides presented activities over the four mycobacterial species that follow the described pattern of DprE1 sensitivity provided important clues that support the hypothesis of the disubstituted derivatives acting as DprE1 inhibitors. The remaining compounds tested did not present a similar spectrum of activity and presented intrinsically low activities against Mtb, which indicates that they either do not inhibit DprE1 or are unable to reach the periplasmic space where DprE1 is located [11,41].

One important aspect of any new antitubercular drug is its activity over dormant inactive cells [42]. We did not test our compounds in any model suitable to assess its activity over non-replicating *M. tuberculosis*, as the probable mechanism of action does not support a relevant activity over dormant cells; however, this hypothesis should be kept open.

The cytotoxicity assessments indicated that despite their nitroaromatic nature, the compounds exhibit low toxicity relative to their antimycobacterial activity. This finding was particularly pronounced in compounds with MIC values below 0.5 µg/mL, where the effective concentrations were substantially lower than the cytotoxic levels, suggesting potential safety in therapeutic contexts. However, in the future, other toxicity studies (*in silico*, *in vitro*, and *in vivo*) must be performed in parallel with the advancement of this family of compounds.

Since no significant impact on macrophage viability was observed at the MIC concentrations used, an infection model with Mtb-infected macrophages was challenged with the most active compounds. Figure 3 allows us to easily distinguish the compounds that lead to the intracellular killing of mycobacteria (decreasing the CFU count number) from the ones that do not. For compounds **10**, **12**, and **13**, pronounced intracellular mycobacterial death was observed, although for compound **12**, a diminished antitubercular activity was achieved. Despite its low MIC value, compound **11** failed to exhibit activity in the macrophage assay. This suggests that factors beyond MIC values are crucial for efficacy in this more complex system. Similarly, compound **12** displayed reduced effectiveness compared to compounds **10** and **13**, raising the possibility that the physicochemical properties related to the length of the alkyl chain adversely affect the antitubercular performance in this experimental context, unlike in standard *in vitro* assays.

From Table 2, it is clear that amide isosteres of the 3,5-dinitrobenzoates have better activity over *M. tuberculosis* than the esters. In previous work, ester analogues of the amides under study were associated with some susceptibility to hydrolysis [24]. However, here it was shown that the generality of amide derivatives was stable in all biological media assessed, and the most active compounds, both *in vitro* and in the macrophage model, were very stable.

Finally, computational studies were performed and aimed at assessing DprE1 as a possible target of action for the most active families of compounds, namely the 3,5-dinitro and 3-nitro-5-trifluoromethyl-substituted derivatives. By comparing our compounds with the literature compounds DNB1 and DNB2, we can observe that, while the score values do not accurately allow us to distinguish highly active (MIC ≤ 2 µg/mL) from less active (MIC > 2 µg/mL) compounds, the distances to the cysteine 387 residue and the FAD cofactor, both to the aromatic ring or the nitro group, allowed us to make this distinction. 

The similarity in distance measurements between our highly active compounds and established DprE1 inhibitors suggests a similar fit within the DprE1 binding pocket, reinforcing DprE1 as a suitable target for these compounds. Metrics such as ΣNDist and ΣCDist help to distinguish between highly active and less active compound families, providing a valuable tool for future research and identifying potential new inhibitors.

It is important to note that these distance metrics do not vary significantly with different alkyl chain lengths because of the enzyme’s broad binding pocket, which can accommodate large, branched chains like those in the natural substrate of DprE1. While this confirms the structural fit is crucial for inhibitory activity, it complicates the ability to rank compounds within families based on effectiveness.

Regarding the correlation between score values and MIC levels, no direct relationship was observed. Instead, score values increased linearly with alkyl chain length and log*P* values, indicating that differences in MIC values are likely due to solubility and absorption challenges rather than binding affinity to DprE1.

## 4. Materials and Methods

Materials. Balanced salt solution, phosphate-buffered saline (PBS), Dulbecco’s modified Eagle’s medium (DMEM), and L-glutamine were purchased from Invitrogen. Sodium dodecyl sulphate (SDS), Triton X-100, benzoic acid, 4-nitrobenzoic acid, 3,5-dinitrobenzoic acid, 3-nitro-5-(trifluoromethyl)benzoic acid, n-butylamine, n-hexylamine, n-octylamine, n-decylamine, n-dodecylamine, n-tetradecylamine, n-hexadecylamine, and trypan blue were purchased from Merck, KGaA (Darmstadt, Germany). Middlebrook 7H10 agar was purchased from Difco (BD Difco, Franklin Lakes, NJ, USA). Nunc Microwell tissue culture plates were purchased from Thermo Fisher Scientific (Waltham, MA, USA). All amides presented were synthesized according to the procedures described in this paper. Compounds were prepared in stock solutions of 40 mg/mL in dimethyl sulfoxide (DMSO—AppliChem Panreac, Darmstadt, Germany). Isoniazid (Merck, KGaA, Darmstadt, Germany) is a first line antibiotic against tuberculosis and was used as a positive control for *M. tuberculosis* killing.

Bacterial strains and cell lines. Bacteria broth culture medium Middlebrook 7H9 and solid culture medium Middlebrook 7H10 were purchased from Difco (BD Difco, Franklin Lakes, NJ, USA). All Mycobacterium spp. were cultivated in Middlebrook’s 7H9 medium supplemented with 10% OADC (oleic acid, albumin, dextrose, catalase) enrichment (BD Difco, Franklin Lakes, NJ, USA), 0.02% glycerol, and 0.05% tyloxapol (Merck, KGaA, Darmstadt, Germany), incubated at 37 °C until exponential growth phase was achieved. *M. tuberculosis* H37Rv (ATCC 27294), *M. avium* (DSM44156), and *M. bovis* BCG (CIP 105050) were used for MIC evaluation. *M. smegmatis* (ATCC607, mc2 155) was used for homogenate preparation and for MIC determination.

Synthesis. 1. Acyl chloride synthesis: A solution of the chosen benzoic acid derivative in thionyl chloride (3 mL per mmol of acid) was refluxed for 5 h, leading to the formation of the desired acyl chloride. The excess thionyl chloride was removed using low pressure evaporation. The product was used without further purification. 2. General protocol to Amide synthesis: A solution of the appropriate acyl chloride (1 eq.) in dichloromethane was added dropwise to a solution of corresponding amine and triethylamine (1.5 eq.) in dichloromethane at 0 °C. When the reaction was complete (as assessed by TLC using hexane:ethyl acetate, 5:1 to 1:1, or ethyl acetate as eluent), the reaction mixture was filtered and the filtrate was washed successively with 10 mL of distilled water and with 15 mL of saturated sodium bicarbonate solution. The dichloromethane solution was subsequently dried, and the solvent evaporated. The residue was purified with column chromatography (silica gel 60) using hexane: ethyl acetate, 5:1 to 1:1, or ethyl acetate as eluent. All compounds were characterized using ^13^C NMR, ^1^H NMR, IR, and HRMS. The purity of the compounds was further tested using HPLC and TLC. Synthesis specifications, yields, and structural data for compounds **11**, **12**, and **18** are described below. Data for the rest of the compounds are available in the Supplementary Materials.

Synthesis of *N*-octyl-3,5-dinitrobenzamide (**11**). Following the described general procedure, 6 mmol (0.837 mL) of 3,5-dinitrobenzoyl chloride was dissolved in DCM (2.5 mL) and added to a solution in DCM (2.5 mL) of 9 mmol (1.480 mL) of n-octylamine and 6 mmol (0.833 mL) of triethylamine. *N*-octyl-3,5-dinitrobenzamide—yellow solid; yield 43%; ^1^H NMR (300 MHz, Chloroform-d) δ 9.18 (t, J = 2.1 Hz, 1H), 8.95 (d, J = 2.1 Hz, 2H), 6.37 (s, 1H), 3.54 (td, J = 7.3, 5.7 Hz, 2H), 1.79–1.61 (m, 2H), 1.50–1.19 (m, 10H), 0.90 (t, J = 7.3 Hz, 3H). ^13^C RMN (300 MHz, Chloroform-d) δ 162.79 (C7), 120.95 (C4), 138.20 (C1), 127.17 (C2 and C6), 148.64 (C3 and C5), 40.89 (C9), 31.76 (C10), 29.44 29.23 29.16 26.98 (C11–C15), 14.05 (C16). Infra-red (IR)—(n, cm^−1^)—1720,43 (C=O. HRMS (ESI+): *m*/*z* calculated for C_15_H_21_N_3_O_5_: 323.34446, found: 324.1562 (M^+^H^+^). Purity by HPLC 99.5%.

Synthesis of *N*-decyl-3,5-dinitrobenzamide (**12**). Following the described general procedure, 6 mmol (0.837 mL) of 3,5-dinitrobenzoyl chloride was dissolved in DCM (2.5 mL) and added to a solution in DCM (2.5 mL) of 9 mmol (1.776 mL) of n-decylamine and 6 mmol (0.833 mL) of triethylamine. *N*-decyl-3,5-dinitrobenzamide—yellow solid; yield 41%; ^1^H NMR (300 MHz, Chloroform-d) δ 9.17 (t, J = 2.0 Hz, 1H), 8.96 (d, J = 2.1 Hz, 2H), 6.47 (s, 1H), 3.54 (td, J = 7.3, 5.7 Hz, 2H), 1.78–1.63 (m, 2H), 1.49–1.18 (m, 14H), 0.89 (t, J = 7.3 Hz, 3H). ^13^C RMN (300 MHz, Chloroform-d) δ 162.79 (C7), 120.94 (C4), 138.20 (C1), 127.18 (C2 and C6), 148.64 (C3 and C5), 40.90 (C9), 31.86 (C10), 29.52 29.44 29.28 26.98 22.65 (C11–C17), 14.08 (C18). Infra-red (IR)—(n, cm^−1^)—1720,43 (C=O). HRMS (ESI+): *m*/*z* calculated for C_17_H_25_N_3_O_5_: 351.39762, found: 352.1869 (M^+^H^+^). Purity by HPLC 99.5%.

Synthesis of *N*-octyl-3-nitro-5-(trifluoromethyl)benzamide (**18**). Following the described general procedure, 6 mmol (0.967 mL) of 3-nitro-5-(trifluoromethyl)benzoyl chloride was dissolved in DCM (2.5 mL) and added to a solution in DCM (2.5 mL) of 9 mmol (1.480 mL) of n-octylamine and 6 mmol (0.833 mL) of triethylamine. *N*-octyl-3-nitro-5-(trifluoromethyl)benzamide—yellow solid; yield 35%; ^1^H NMR (300 MHz, Chloroform-d) δ 8.76 (br. s, 1H), 8.60 (br. s, 1H), 8.41 (br. s, 1H), 6.40 (s, 1H), 3.50 (td, J = 7.3, 5.7 Hz, 2H), 1.75–1.55 (m, 2H), 1.48–1.16 (m, 10H), 0.88 (t, J = 7.3 Hz, 3H). ^13^C RMN (300 MHz, Chloroform-d) δ 163,77 (C7), 120.64 (C4), 137,63 (C1), 132.90 (q, J_CF_ = 34.7 Hz, C5), 124,75 (C6), 129.97 (C2), 123,04 (CF_3_), 148.36 (C3), 40.77 (C9), 31.76 (C10), 29.44 29.23 29.16 26.97 22.60 (C11–C15), 14.03 (C16). Infra-red (IR)—(n, cm^−1^)—1720.43 (C=O). HRMS (ESI+): *m*/*z* calculated for C_16_H_21_F_3_N_2_O_3_: 346.34482, found: 347.1496 (M^+^H^+^). Purity by HPLC 98.7%.

Compound characterization. ^1^H NMR and ^13^C-NMR spectra were recorded on a Bruker Ultra-Shield 300 MHz spectrometer in the indicated solvent; chemical shifts are reported in parts per million (ppm), relative to tetramethylsilane (TMS). The spectra were referenced to the solvent peak and coupling constants (*J*) are quoted in hertz (Hz). The IR spectra were recorded on a 400 FTIR Nicolet Impact spectrometer between 4000 and 400 cm^−1^. High-resolution mass spectra (HRMS) were obtained on a Bruker Impact II quadrupole time-of-flight mass spectrometer (Bruker Daltonics, Billerica, MA, USA), and *m*/*z* values are reported in Daltons.

HPLC analysis: HPLC determinations were performed in a HPLC Hitachi LaChrom Ultra system comprising two Hitachi L-2160U pumps, a Hitachi UV-L-2400U detector, a Hitachi L-2200U auto sampler, a Hitachi L-2300 column oven, a Merck-Hitachi D-2500 integrator, and a Merck RP-8 column. The eluant was a mixture of acetonitrile (60% to 80%) and aqueous phosphate buffer with 5% of KH_2_PO_4_/H_3_PO_4_ 0.025M (40% to 20%). The flow rate was 1 mL min^−1^ and the wavelength was set to 230 nm. All quantifications were evaluated using calibration curves from stock solutions. Purity by HPLC was determined using a Merck RP-8 column % 50% ACN in water for compounds **1**, **2**, **3**, **5**, **7**, **9**, **10**, **11**, **16**, **17**, and **18** and 70% acetonitrile in water for the other compounds.

Mycobacterial homogenate preparation: A crude whole mycobacterial homogenate was prepared according to reference [20]. A culture of exponentially growing *M. smegmatis* ATCC607 variant mc^2^ 155 with an O.D.600 nm of 0.8–1.0 was harvested with centrifugation at T = 4 °C for 10 min, washed, and re-suspended in pH = 7.4 phosphate-buffered saline (PBS) (25 mL for each 750 mL of the initial growing broth). The bacterial homogenate was prepared using an ultrasound probe with a sequence of five cycles of 2 min each. Afterwards, the homogenate was divided in 1 mL portions and kept at −80 °C until use. Total protein concentration was 1.4 mg mL^−1^.

Stability studies. Conditions of incubations and preparation of samples: In all stability assays, the initial concentration of the compound under study was 5 × 10^−4^ M. All incubations were carried out at pH 7.4 and 37 °C under agitation using PBS as diluting agent. The benzamides were added from 2.5 × 10^−2^ M acetonitrile stock solutions. After incubation, aliquots of 50 μL were taken into vials, processed as described below, injected into the HPLC, and analyzed for quantification of the corresponding acid and remaining benzamide. All quantifications were performed using calibration curves. Stability in buffer. A total of 1600 µL pH 7.4 phosphate-buffered saline (PBS), 360 µL ACN, and 40 µL of a stock solution of the compound in ACN were mixed in a 2 mL vial. The solution was incubated at 37 °C and 50 µL aliquots were removed, mixed with 450 µL of ACN: H_2_O 1:1, and analyzed using HPLC. Plasma stability. Pooled human plasma (640 µL), pH 7.4 phosphate-buffered saline (160 µL), and 16 µL of a 2.5 × 10^−2^ M stock solution of the compound in ACN were mixed in a vial. The suspensions were incubated at 37 °C and 50 µL aliquots were removed, mixed with 450 µL of ACN: ZnSO_4_ 1% 1:1, and centrifuged for 10 min at 15,000 rpm. The supernatant was then removed and analyzed using HPLC. Homogenate stability. A total of 948 µL pH 7.4 phosphate buffer, 32 µL of *M. smegmatis* homogenate, and 20 µL of a 2.5 × 10^−2^ M stock solution of the compound in ACN were mixed in a vial. The solution was incubated at 37 °C and 50 µL aliquots were removed, mixed with 450 µL of ACN: H_2_O 1:1, and centrifuged for 10 min at 15,000 rpm. The supernatant was then removed and analyzed using HPLC.

Determination of the Minimum Inhibitory Concentration (MIC) and Minimum Bactericidal Concentration (MBC): The MICs were determined using the broth microdilution method in 96-well plates. Briefly, *M. tuberculosis* bacterial cultures in exponential growth phase were collected using centrifugation, washed with PBS, and re-suspended in fresh culture medium. Clumps of bacteria were removed with ultrasonic treatment of the bacteria suspension in an ultrasonic water bath for 5 min, followed by a low-speed centrifugation (500× *g*) for 2 min. Single cell suspension was verified using microscopy. The microplates containing a bacterial suspension corresponding to approximately 10^5^ colony-forming units per ml were incubated with the selected concentrations of the compounds. Every other day, the optical density of the wells was measured in a Tecan M200 spectrophotometer, following 30 s of orbital agitation. These values were used to produce the growth curves. At the 10th day of incubation, the MIC was determined, corresponding to the concentration with no visible turbidity. Optical density measurements were taken until the 15th day of incubation. The MBC values were determined using an established methodology [43]. Briefly, following MIC determination, the bacterial samples were recovered from the MIC test microplates and plated in 7H10 + OADC solid medium. The MBC was determined following 3 weeks of incubation, corresponding to the concentration of compound that produced no colonies on the solid medium. Bacteria treated with DMSO solvent at the same proportions as present during the compound tests were used as a control. Isoniazid was used as a positive control for bacteria killing and assay validation was performed following EUCAST guidelines (MIC = [0.03, 0.12] μg/mL). All MIC and MBC results presented comprise the mode value of a minimum of triplicate experiments.

Determination of macrophage viability after treatment with compounds: The human monocytic cell line THP-1 (ATCC TIB202) was used to determine the effect of the compounds on cell viability. The cells were grown in RPMI 1640 (BD Difco, Franklin Lakes, NJ, USA), supplemented with 10% fetal bovine serum (FBS; BD Difco, Franklin Lakes, NJ, USA), 10 mM HEPES (BD Difco, Franklin Lakes, NJ, USA), 1 mM sodium pyruvate, and maintained at 37 °C with 5% CO_2_. Differentiation of THP-1 monocytes into macrophages was induced for 48 h with 20 nM phorbol 12-myristate 13-acetate (PMA), following a 24 h resting period without PMA. Differentiated macrophages in 96-well plates, 5 × 10^4^ cells per well, were treated with the compounds. After three days of treatment, cell viability was determined using PrestoBlue (Invitrogen, Carlsbad, CA, USA) following the manufacturer’s indications. Briefly, the cells were washed with PBS and incubated with PrestoBlue 10% (*v*/*v*) in cell culture medium. After 4 h of incubation, the fluorescence of each well was measured in a Tecan M200 spectrophotometer (Em: 560 nm/Ex: 590 nm). Viability was calculated relatively to non-treated cells. Cells treated with DMSO solvent at the same proportions were used as a control. Puromycin was used as a positive control for cell death.

Macrophage Infection and intracellular bacteria killing following compounds treatment: Before infection, *M. tuberculosis* was cultivated for seven days at 37 °C and 5% CO_2_ until the exponential growth phase was reached. Bacterial suspensions were centrifuged and washed in phosphate-buffered saline (PBS) and resuspended in macrophage culture medium without antibiotics. Clumps of bacteria in the suspension were disrupted using an ultrasonic bath treatment for 5 min and removed using centrifugation at a low speed of 500× *g* for 1 min. The obtained single-cell suspension was verified through fluorescence microscopy and quantified by measuring the optical density at 600 nm. Then, the infection was performed with a multiplicity of infection (MOI) of 1 bacterium per macrophage for 3 h at 37 °C and 5% CO_2_. Following this incubation period, the cells were washed with PBS and the compounds (0.15 μg/mL) were added in fresh, complete medium. DMSO solvent was used as a negative control and isoniazid (0.15 μg/mL) was used as a positive control for bacteria killing. At the specified time-points, macrophages were disrupted using 0.05% Igepal and the resulting bacterial suspension was serial-diluted and plated in 7H10 OADC agar plates. Micro-colonies were counted following approximately 2 weeks of incubation at 37 °C.

Molecular docking: Three-dimensional models of the compounds under study were built and optimized (MMFF94forcefield) using the open-source cheminformatics toolkit RDK [44]. Meeko library (https://github.com/forlilab/Meeko, (accessed on 9 October 2023) was used to obtain the corresponding PDBQT files, allowing all available torsions to rotate freely. The receptor structure was retrieved from the RCSB Protein Data Bank entry 4P8L (PDB: 4P8L), correspondent to the crystal structure of *M. tuberculosis* DprE1 in complex with the non-covalent inhibitor Ty36c, and only the atoms pertaining to the chain A of the protein were considered. A PDBQT file of the receptor was obtained using AutoDockTools [45], keeping only the polar hydrogen atoms. Molecular docking simulations were performed using AutoDock Vina 1.2.5 [46,47]. Two search spaces of 22 Å^3^ and 30 Å^3^ were used, centered on the position of the ligand from the crystallographic structure. For each compound, 5 independent docking runs were performed to increase sampling, using 3 scoring functions for pose selection: Vina, Vinardo, and AutoDock. All poses were then sorted according to each of the scoring functions, selecting the lowest-energy conformations for each docking run and each compound. The coordinates of the 5 top poses for each compound were further used to calculate the distance from the N atom of their nitro groups and the center of their aromatic ring moiety to two atoms of the receptor: the S atom in the cysteine 387 residue and the N atom in the center portion of the isoalloxazine ring of the FAD cofactor that is in the binding pocket region (Figure 4B).

## 5. Conclusions

The synthesis of a library of nitro-substituted alkyl benzamides allowed us to enhance the understanding of the role of alkyl groups in the antitubercular activity of the compounds.

The results here discussed show that high antitubercular activities are obtained with di-substitution of the aromatic moiety as well as an alkyl chain length of eight to ten carbon atoms. Regarding the alkyl chain length, a parabolic influence on activity was observed for the most active family of compounds. Also, we demonstrated that such compounds have good profiles of activity in infection models, comparable to INH, a known antitubercular drug.

While we do not have definitive proof that our compounds inhibit DprE1, the evidence gathered presents a compelling case. The structural resemblance of these compounds to known inhibitors, combined with susceptibility data from multiple mycobacterial species, and computational studies, support the hypothesis that DprE1 is a likely target. 

The computational studies indicate that a good fit in the binding pocket is associated with a good activity, but the physical properties of compounds, such as the log*P* value, must also be taken into account, especially for their impact on solubility and their capacity for crossing the cell wall (but not the membrane) into the cell periplasm where DprE1 is located [13]. 

Our results indicate that N-alkyl chains with a length of 10 carbons significantly enhance the activity of compounds, particularly within the 3,5-dinitro family, which emerges as the most promising group. Compound **12** exemplifies this finding. This study underscores the potential of incorporating flexible alkyl chains into the DNB class of compounds, offering a strategic approach to designing new active derivatives that leverage the advantages of such structural modifications.

## 6. Patents

The current work led to European Patent Application EP22199251. (EPO). Luis Filipe Vicente Constantino, João Pedro Almeida Pais, Tiago Alexandre Duarte Delgado, Olha Antoniuk, Elsa Maria Ribeiro Dos Santos Anes, and David Alexandre Rodrigues Pires. (2022). Benzoic acid derivatives, methods and uses thereof.

## Figures and Tables

**Table 1:** Library of compounds under study and their corresponding predicted log*P* values, as well as their antitubercular activity against *M. tuberculosis*, expressed as MIC and MBC values, which are the mode value of triplicate experiments.

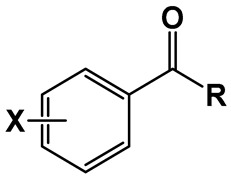					
Compound	X	R	Log*P* ^1^	MIC (µg/mL)	MBC (µg/mL)
**1**	H	NH(CH_2_)_3_CH_3_	2.45	256	256
**2**		NH(CH_2_)_5_CH_3_	3.52	64	64
**3**		NH(CH_2_)_7_CH_3_	4.58	32	32
**4**		NH(CH_2_)_11_CH_3_	6.7	>256	>256
**5**	4-NO_2_	NH(CH_2_)_3_CH_3_	2.62	128	256
**6**		NH(CH_2_)_5_CH_3_	3.68	32	64
**7**		NH(CH_2_)_7_CH_3_	4.74	128	256
**8**		NH(CH_2_)_11_CH_3_	6.87	512	>1024
**9**	3,5-NO_2_	NH(CH_2_)_3_CH_3_	2.21	0.5	0.5
**10**		NH(CH_2_)_5_CH_3_	3.28	0.031	0.031
**11**		NH(CH_2_)_7_CH_3_	4.34	0.016	0.031
**12**		NH(CH_2_)_9_CH_3_	5.4	0.016	0.016
**13**		NH(CH_2_)_11_CH_3_	6.46	0.063	0.063
**14**		NH(CH_2_)_13_CH_3_	7.53	0.125	0.25
**15**		NH(CH_2_)_15_CH_3_	8.59	2	2
**16**	3-NO_2_-5-CF_3_	NH(CH_2_)_3_CH_3_	3.52	2	2
**17**		NH(CH_2_)_5_CH_3_	4.58	0.5	0.5
**18**		NH(CH_2_)_7_CH_3_	5.65	0.016	0.031
**19**		NH(CH_2_)_9_CH_3_	6.71	0.5	0.5
**20**		NH(CH_2_)_11_CH_3_	7.77	0.5	0.5

^1^ Log*P* values were predicted by the software ALOGPS 2.1 [28].

**Table 2:** Susceptibility of *M. tuberculosis*, *M. bovis* BCG, *M. avium*, and *M. smegmatis* to synthesized and control compounds, expressed as MIC (µg/mL) and MBC (µg/mL) values. DNB1, DNB2, PAS, and INH were used as internal standards.

Compounds	*M. tuberculosis*		*M. bovis* BCG		*M. smegmatis*		*M. avium*	
	MIC	MBC	MIC	MBC	MIC	MBC	MIC	MBC
DNB1	0.031	0.031	0.063		0.5	1	32	>128
DNB2	0.031	0.063	0.125		0.5	1	64	>128
PAS	≤0.063	≤0.063	≤0.008	≤0.008	1024	>1024	1024	1024
INH	0.05	0.05	0.025	0.025	8	>25.6	>25.6	>25.6
**3**	32	32	32	32	128	128	128	256
**7**	128	256	128	512	1024	1024	1024	1024
**9**	0.5	0.5	0.5	1	4	4	64	512
**10**	0.063	0.063	0.25	0.25	1	1		
**11**	0.016	0.031	0.016	0.016	0.25	4	512	>512
**12**	0.016	0.016	0.063	0.063	1	1	>512	>512
**13**	0.031	0.063	0.083	0.166	0.667	>2.664	>512	>512
**14**	0.125	0.25	0.25	0.25	1	4		
**15**	2	2	2	4	32	>64		
O9	32	128	16	16	32	64	128	256
O10	32	4	8	8	8	8		
O11	8	8	2	4	4	4	>1024	>1024
O12	2	4	8	8	16	32	>512	>512
O13	8	16	8	8	>512	>512	>512	>512
O14	16	>1024	8	8	>1024	>1024	>512	>512
O15	>1024	>1024	128	256	>1024	>1024	>512	>512

**Table 3:** Results obtained from the cytotoxicity assays, represented by LC_50_ values (μg/mL), the MIC values (μg/mL), and the ratio LC_50_/MIC.

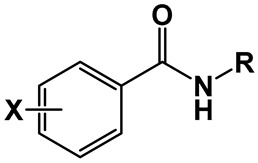					
Compounds	X	R	MIC	LC_50_	LC_50_/MIC
			(μg/mL)	(μg/mL)	
**3**	H	C_8_H_17_	32	188	6
**7**	4-NO_2_	C_8_H_17_	128	308	2
**9**	3,5-dNO_2_	C_4_H_9_	0.5	26	52
**10**		C_6_H_13_	0.031	361	11,645
**11**		C_8_H_17_	0.016	97	6063
**12**		C_10_H_21_	0.016	53	3313
**13**		C_12_H_25_	0.063	72	1143
**14**		C_14_H_29_	0.125	330	2640
**15**		C_16_H_32_	2	n/d	-
**16**	3-NO_2_-5-CF_3_	C_4_H_9_	2	41	21
**17**		C_6_H_13_	0.5	23	46
**18**		C_8_H_17_	0.016	14	875
**19**		C_10_H_21_	0.5	53	106
**20**		C_12_H_25_	0.5	113	226
Puromycin			-	0.35	-

**Table 4:** Results obtained from the stability assays in human plasma and mycobacterial homogenate. ND refers to the K_obs_ that could not be accurately calculated as the hydrolysis rate was too slow. % (72 h) or % (48 h) represents the percentage of hydrolysis after a 72- or 48-h incubation period, respectively, and allows for a comparison of relative stability.

Compound	Human Plasma		Mycobacterium Homogenate	
	k_obs_ × 100 (h^−1^)	% (72 h)	k_obs_ × 100 (h^−1^)	% (48 h)
**1**	ND	0	ND	3
**2**	-	-	ND	2
**3**	ND	0	ND	2
**4**	ND	0	ND	0
**5**	ND	32	0.91 ± 0.07	35
**6**	-	-	0.80 ± 0.05	32
**7**	ND	1	ND	2
**8**	ND	1	ND	3
**9**	ND	0	3.03 ± 0.20	77
**10**	-	-	0.84 ± 0.01	33
**11**	ND	0	ND	5
**12**	-	-	ND	2
**13**	ND	0	ND	2
**14**	-	-	ND	2
**15**	-	-	ND	0
**16**	ND	0	1.09 ± 0.07	41
**17**	-	-	ND	14
**18**	ND	0	ND	11
**19**	ND	0	ND	3
**20**	ND	0	ND	2

ND (not determined); “-” (not assayed).

**Table 5:** Values of the distances obtained from the analysis of the best binding poses (AutoDock) of the compounds in study and their correspondent scores (kcal/mol) and MIC (µg/mL) values. ND_Cys387_ and ND_FAD_ refers to the distance between the nitro group to the cysteine 387 and FAD residues, respectively. Similarly, CD_Cys387_ and CD_FAD_ refers to the distance between the center of the aromatic moiety to the cysteine 387 and FAD residues, respectively. ΣNDist and ΣCDist refer to the sum of the distances to the nitro group and the aromatic center, respectively.

Compound	MIC (µg/mL)	ND_Cys387_(Å)	ND_FAD_(Å)	ΣNDist	CD_Cys387_(Å)	CD_FAD_(Å)	ΣCDist	Score_Autodock_
DNB1	0.016	3.562	3.908	7.470	5.575	5.069	10.645	−8.487
DNB2	0.031	3.531	4.008	7.539	4.989	3.352	8.341	−8.639
**1**	256	-	-	-	9.444	13.128	22.572	−5.495
**2**	64	-	-	-	11.572	11.664	23.235	−6.021
**3**	32	-	-	-	9.523	13.200	22.723	−6.672
**4**	>256	-	-	-	12.150	11.659	23.809	−7.507
**5**	128	12.765	12.345	25.110	12.079	12.044	24.124	−6.431
**6**	32	12.784	12.322	25.106	11.841	11.938	23.779	−7.091
**7**	128	12.702	12.286	24.989	11.683	11.841	23.525	−7.419
**8**	512	5.079	3.862	8.941	12.218	12.185	24.403	−8.261
**9**	0.5	3.371	3.814	7.185	5.015	4.935	9.950	−7.313
**10**	0.031	3.440	3.745	7.185	4.949	5.085	10.034	−7.688
**11**	0.016	3.421	3.927	7.348	5.461	5.221	10.682	−8.305
**12**	0.016	3.370	3.760	7.131	4.806	4.813	9.619	−8.584
**13**	0.063	3.484	3.667	7.151	4.944	4.989	9.932	−9.064
**14**	0.125	3.471	3.698	7.169	4.928	5.064	9.992	−9.356
**15**	2	3.439	3.731	7.170	5.438	5.011	10.448	−9.706
**16**	2	6.048	7.087	13.135	5.417	4.958	10.374	−6.672
**17**	0.5	5.432	4.410	9.843	5.076	3.471	8.547	−7.161
**18**	0.016	5.944	4.447	10.392	5.108	3.535	8.643	−7.600
**19**	0.5	5.907	4.482	10.390	5.370	4.941	10.312	−8.057
**20**	0.5	5.225	3.915	9.140	4.754	3.387	8.141	−8.522

## Data Availability

Data available within the article.

## Supplementary Materials

The following supporting information can be downloaded at: https://www.mdpi.com/article/10.3390/ph17050608/s1, Synthesis specifications and characterization of compounds.
