# Supplementary material for: Synthesis, Activity, Toxicity, and In Silico Studies of New Antimycobacterial N-Alkyl Nitrobenzamides
Source: Pharmaceuticals (Basel). 2024 May 9;17(5):608. doi: 10.3390/ph17050608 (PMC11124399; doi:10.3390/ph17050608)

## Supplementary materials

# Synthesis, Activity, Toxicity, and In Silico Studies of New Antimycobacterial *N*-Alkyl Nitrobenzamides

**Pais** ***et al.***

**Synthesis.** 1.Acyl chloride synthesis: A solution of the chosen benzoic acid derivative in thionyl chloride (3 mL per mmol of acid) was refluxed for 5 h, leading to the formation of the desired acyl chloride. The excess thionyl chloride was removed by low pressure evaporation. The product was used without further purification. 2.Amide synthesis: A solution of the appropriate acyl chloride (1 eq.) in dichloromethane was added dropwise to a solution of corresponding amine and triethylamine (1.5 eq.) in dichloromethane at 0°C. When the reaction was complete (as assessed by TLC using hexane:ethyl acetate, 5:1 to 1:1,or ethyl acetate as eluent) the reaction mixture was filtered and the filtrate washed successively with 10 mL of distilled water and with 15 mL of saturated sodium bicarbonate solution. The dichloromethane solution was subsequently dried, and the solvent evaporated. The residue was purified by column chromatography (silica gel 60) using hexane: ethyl acetate, 5:1 to 1:1, or ethyl acetate as eluent.

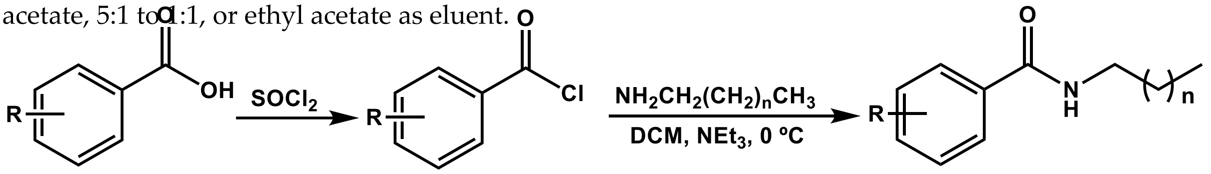


**Figure S1**. Synthetic scheme for the synthesis of the *N*-alkyl nitrobenzamides under study

*Synthesis of N-butyl*-benzamide **(1)**. Following the described general procedure, 6 mmol (0,695 mL) of benzoyl chloride were dissolved in DCM (2,5 mL) and added to a solution in DCM (2,5 mL) of 9 mmol (0,885 mL) of n-butylamine and 6 mmol (0,833 mL) of triethylamine. *N*-butyl-benzamide - yellow oil; Yield 84%; $^1$H NMR (300 MHz, Chloroform-d) δ 7.82 – 7.70 (m, 2H), 7.56 – 7.34 (m, 3H), 6.15 (s, 1H), 3.46 (td, J = 7.1, 5.7 Hz, 2H), 1.66 – 1.54 (m, 2H), 1.50 – 1.33 (m, 2H), 0.96 (t, J = 7.3 Hz, 3H). $^{13}$C RMN (300 MHz, Chloroform-d) δ 167.64 (C7), 131.23 (C4), 134.87 (C1), 128.46 (C2 and C6), 126.89 (C3 and C5), 39.82 (C9), 31.72 (C10), 20.15 (C11), 13.77 (C12). Infra-red (IR) - (n, cm-1) - 1720,43 (C=O). HRMS (ESI$^+$): *m*/*z* calculated for $C_{11}H_{15}NO$: 177.24294, found: 178.1227 (M+H$^+$). Purity by HPLC 99.9%.

Synthesis of *N*-hexyl-benzamide **(2).** Following the described general procedure, 6 mmol (0,695 mL) of benzoyl chloride were dissolved in DCM (2,5 mL) and added to a solution in DCM (2,5 mL) of 9 mmol (1,182 mL) of n-hexylamine and 6 mmol (0,833 mL) of triethylamine. *N*-hexyl-benzamide - yellow solid ; Yield 85%; $^1$H NMR (300 MHz, Chloroform-d) δ 7.83 – 7.69 (m, 2H), 7.55 – 7.35 (m, 3H), 6.08 (s, 1H), 3.46 (td, J = 7.1, 5.7 Hz, 2H), 1.70 – 1.56 (m, 2H), 1.46 – 1.24 (m, 6H), 0.90 (t, J = 7.3 Hz, 3H). $^{13}$C RMN (300 MHz, Chloroform-d) δ 167.53 (C7), 131.24 (C4), 134.91 (C1), 128.49 (C2 and C6), 126.86 (C3 and C5), 40.12 (C9), 31.51 (C10), 29.64 26.68 22.56 (C11-C13), 14 (C14). Infra-red (IR) - (n, cm-1) - 1720,43 (C=O). HRMS (ESI$^+$): *m*/*z* calculated for $C_{13}H_{19}NO$: 205.2961, found: 206.1535 (M+H$^+$). Purity by HPLC 99,5%.

Synthesis of *N*-octyl-benzamide (**3**). Following the described general procedure, 6 mmol (0,695 mL) of benzoyl chloride were dissolved in DCM (2,5 mL) and added to a solution in DCM (2,5 mL) of 9

mmol (1,480 mL) of n-octylamine and 6 mmol (0,833 mL) of triethylamine. *N*-octyl-benzamide - white solid ; Yield 80%; $^{1}$H NMR (300 MHz, Chloroform-d) δ 7.82 – 7.71 (m, 2H), 7.57 – 7.36 (m, 3H), 6.11 (s, 1H), 3.47 (td, J = 7.2, 5.6 Hz, 2H), 1.74 - 1.53 (m, 2H), 1.47 – 1.17 (m, 10H), 0.90 (t, J = 7.3 Hz, 3H). $^{13}$C RMN (300 MHz, Chloroform-d) δ 167.53 (C7), 131.24 (C4), 134.92 (C1), 128.49 (C2 and C6), 126.86 (C3 e C5), 40.13 (C9), 31.79 (C10), 29.68 29.29 27.02 22.63 (C11-C15), 14.07 (C16). Infra-red (IR) - (n, cm-1) - 1720,43 (C=O). HRMS (ESI$^+$): *m/z* calculated for $C_{15}H_{23}NO$: 233.34926, found: 234.1849 (M+H$^+$). Purity by HPLC 99,8%.

Synthesis of *N*-dodecyl-benzamide **(4).** Following the described general procedure, 6 mmol (0,695 mL) of benzoyl chloride were dissolved in DCM (2,5 mL) and added to a solution in DCM (2,5 mL) of 9 mmol (2.074 mL) of n-dodecylamine and 6 mmol (0,833 mL) of triethylamine. *N*-dodecyl-benzamide - yellow solid ; Yield 78%; $^1$H NMR (300 MHz, Chloroform-d) δ 7.82 – 7.72 (m, 2H), 7.57 – 7.38 (m, 3H), 6.11 (s, 1H), 3.47 (td, J = 7.2, 5.7 Hz, 2H), 1.71 – 1.53 (m, 2H), 1.47 – 1.17 (m, 18H), 0.90 (t, J = 7.3 Hz, 3H). $^{13}$C RMN (300 MHz, Chloroform-d) δ 167.50 (C7), 131.25 (C4), 134.92 (C1), 128.51 (C2 and C6), 126.84 (C3 and C5), 40.13 (C9), 31.91 (C10), 29.69 29.64 29.34 27.02 22.68 (C11-C19), 14.10 (C20). Infra-red (IR) - (n, cm-1) - 1720,43 (C=O). HRMS (ESI$^+$): *m/z* calculated for $C_{19}H_{31}NO$: 289.45558, found: 290.2472 (M+H$^+$). Purity by HPLC 98,9%.

Synthesis of *N*-butyl-4-nitrobenzamide **(5).** Following the described general procedure, 6 mmol (0,766 mL) of 4-nitrobenzoyl chloride were dissolved in DCM (2,5 mL) and added to a solution in DCM (2,5 mL) of 9 mmol (0,885 mL) of n-butylamine and 6 mmol (0,833 mL) of triethylamine. *N*-butyl-4-nitrobenzamide - white solid ; Yield 93%; $^1$H NMR (300 MHz, Chloroform-d) δ 8.30 (ddd, Jo= 9 and Jm = 1,8; 2,1 Hz, 2H, C3-H and C5-H), 7.93 (ddd, Jo= 9 and Jm = 1,8; 2,1 Hz, 2H, C2-H e C6-H), 6.19 (s, 1H), 3.50 (td, J = 7.2, 5.7 Hz, 2H), 1.71 - 1.56 (m, 2H), 1.53 - 1.34 (m 2H), 0.99 (t, J = 7.3 Hz, 3H). $^{13}$C RMN (300 MHz, Chloroform-d) δ 165.54 (C7), 149.47 (C4), 140.47 (C1), 128.09 (C2 and C6), 123.74 (C3 and C5), 40.17 (C9), 31.56 (C10), 20.13 (C11), 13.72 (C12). Infra-red (IR) - (n, cm-1) - 1720,43 (C=O). HRMS (ESI$^+$): *m/z* calculated for $C_{11}H_{14}N_2O_3$: 222.24054, found: 223.1070 (M+H$^+$). Purity by HPLC 99,7%.

Synthesis of *N*-hexyl-4-nitrobenzamide **(6).** Following the described general procedure, 6 mmol (0,766 mL) of 4-nitrobenzoyl chloride were dissolved in DCM (2,5 mL) and added to a solution in DCM (2,5 mL) of 9 mmol (1,182 mL) of n-hexylamine and 6 mmol (0,833 mL) of triethylamine. *N*-hexyl-4-nitrobenzamide - white solid ; Yield 40%; $^1$H NMR (300 MHz, Chloroform-d) δ 8.31 (ddd, Jo= 9 e Jm = 1,8; 2,1 Hz, 2H, C3-H and C5-H), 7.93 (ddd, Jo= 9 and Jm = 1,8; 2,1 Hz, 2H, C2-H and C6-H), 6.14 (s, 1H), 3.50 (td, J = 7.2, 5.7 Hz, 2H), 1.72 – 1.59 (m, 2H), 1.50 – 1.22 (m, 6H), 0.92 (t, J = 7.3 Hz, 3H). $^{13}$C RMN (300 MHz, Chloroform-d) δ 165.49 (C7), 149.49 (C4), 140.46 (C1), 128.07 (C2 and C6), 123.77 (C3 and C5), 40.48 (C9), 31.45 (C10), 29.49 26.63 22.53 (C11-C13), 13.98 (C14). Infra-red (IR) - (n, cm-1) - 1720,43 (C=O). HRMS (ESI$^+$): *m/z* calculated for $C_{13}H_{18}N_2O_3$: 250.2937, found: 251.1385 (M+H$^+$). Purity by HPLC 99,1%.

Synthesis of *N*-octyl-4-nitrobenzamide **(7).** Following the described general procedure, 6 mmol (0,766 mL) of 4-nitrobenzoyl chloride were dissolved in DCM (2,5 mL) and added to a solution in DCM (2,5 mL) of 9 mmol (1,480 mL) of n-octylamine and 6 mmol (0,833 mL) of triethylamine. *N*-octyl-4-nitrobenzamide - white solid ; Yield 40%; $^1$H NMR (300 MHz, Chloroform-d) δ 8.29 (ddd, Jo= 9 and Jm = 1,8; 2,1 Hz, 2H, C3-He C5-H), 7.91 (ddd, Jo= 9 and Jm = 1,8; 2,1 Hz, 2H, C2-H and C6-H), 6.17 (s, 1H), 3.48 (td, J = 7.2, 5.7 Hz, 2H), 1.74 – 1.54 (m, 2H), 1.47 – 1.17 (m, 12H), 0.89 (t, J = 7.3 Hz, 3H). $^{13}$C RMN (300 MHz, Chloroform-d) δ 165.49 (C7), 149.49 (C4), 140.46 (C1), 128.08 (C2 and C6), 123.76 (C3 and C5), 40.48 (C9), 31.76 (C10), 29.52 29.24 26.98 22.61 (C11-C15), 14.05 (C16). Infra-red (IR) - (n, cm-1) - 1720,43 (C=O). HRMS (ESI$^+$): *m/z* calculated for $C_{15}H_{22}N_2O_3$: 278.34686, found: 279.1692 (M+H$^+$). Purity by HPLC 98,7%.

Synthesis of *N*-dodecyl-4-nitrobenzamide **(8).** Following the described general procedure, 6 mmol (0,766 mL) of 4-nitrobenzoyl chloride were dissolved in DCM (2,5 mL) and added to a solution in DCM (2,5 mL) of 9 mmol (2.074 mL) of n-dodecylamine and 6 mmol (0,833 mL) of triethylamine.*N*-

dodecyl-4-nitrobenzamide - white solid; Yield 50%; $^1$H NMR (300 MHz, Chloroform-d) δ 8.30 (ddd, Jo= 9 and Jm = 1,8; 2,1 Hz, 2H, C3-H and C5-H), 7.93 (ddd, Jo= 9 and Jm = 1,8; 2,1 Hz, 2H, C2-H and C6-H), 6.17 (s, 1H), 3.49 (td, J = 7.2, 5.7 Hz, 2H), 1.74 - 1.54 (m, 4H), 1.46 – 1.17 (m, 16H), 0.89 (t, J = 7.3 Hz, 3H). $^{13}$C RMN (300 MHz, Chloroform-d) δ 165.54 (C7), 149.56 (C4), 140.47 (C1), 128.08 (C2 and C6), 123.76 (C3 and C5), 40.68 (C9), 31.64 (C10), 26.84 (C11-C15), 14.05 (C20). Infra-red (IR) - (n, cm-1) - 1720,43 (C=O). HRMS (ESI$^+$): *m/z* calculated for $C_{19}H_{30}N_2O_3$: 334.45318, found: 335.2551 (M+H$^+$). Purity by HPLC 97,0%.

Synthesis of *N*-butyl-3,5-dinitrobenzamide **(9).** Following the described general procedure, 6 mmol (0,837 mL) of 3,5-dinitrobenzoyl chloride were dissolved in DCM (2,5 mL) and added to a solution in DCM (2,5 mL) of 9 mmol (0,885 mL) of n-butylamine and 6 mmol (0,833 mL) of triethylamine. *N*-butyl-3,5-dinitrobenzamide - white solid; Yield 47%; $^1$H NMR (300 MHz, Chloroform-d) δ 9.18 (t, J = 2.1 Hz, 1H), 8.96 (d, J = 2.1 Hz, 2H), 6.40 (s, 1H), 3.56 (td, J = 7.3, 5.7 Hz, 2H), 1.75 – 1.63 (m, 2H), 1.54 – 1.38 (m, 2H), 1.01 (t, J = 7.3 Hz, 3H). $^{13}$C RMN (300 MHz, Chloroform-d) δ 162.87 (C7), 120.95 (C4), 138.18 (C1), 127.21 (C2 and C6), 148.62 (C3 and C5), 40.58 (C9), 31.45 (C10), 20.13 (C11), 13.70 (C12). Infra-red (IR) - (n, cm-1) - 1720,43 (C=O). HRMS (ESI$^+$): *m/z* calculated for $C_{11}H_{13}N_3O_5$: 267.23814, found: 268.0888 (M+H$^+$). Purity by HPLC 98,7%.

Synthesis of *N*-hexyl-3,5-dinitrobenzamide **(10).** Following the described general procedure, 6 mmol (0,837 mL) of 3,5-dinitrobenzoyl chloride were dissolved in DCM (2,5 mL) and added to a solution in DCM (2,5 mL) of 9 mmol (1,182 mL) of n-hexylamine and 6 mmol (0,833 mL) of triethylamine. *N*-hexyl-3,5-dinitrobenzamide - yellow solid ; Yield 59%; $^1$H NMR (300 MHz, Chloroform-d) δ 9.17 (t, J = 2.1 Hz, 1H), 8.96 (d, J = 2.1 Hz, 2H), 6.42 (s, 1H), 3.55 (td, J = 7.3, 5.7 Hz, 2H), 1.78 – 1.62 (m, 2H), 1.51 – 1.21 (m, 6H), 0.91 (t, J = 7.3 Hz, 3H). $^{13}$C RMN (300 MHz, Chloroform-d) δ 162.79 (C7), 120.95 (C4), 138.20 (C1), 127.17 (C2 and C6), 148.64 (C3 and C5), 40.89 (C9), 31.43 (C10), 29.40 26.63 22.52 (C11-C13), 13.97 (C14). Infra-red (IR) - (n, cm-1) - 1720,43 (C=O). HRMS (ESI$^+$): *m/z* calculated for $C_{13}H_{17}N_3O_5$: 295.2913, found: 296.1235 (M+H$^+$). Purity by HPLC 99,7%.

Synthesis of *N*-dodecyl-3,5-dinitrobenzamide **(13).** Following the described general procedure, 6 mmol (0,837 mL) of 3,5-dinitrobenzoyl chloride were dissolved in DCM (2,5 mL) and added to a solution in DCM (2,5 mL) of 9 mmol (2.074 mL) of n-dodecylamine and 6 mmol (0,833 mL) of triethylamine. *N*-dodecyl-3,5-dinitrobenzamide - yellow solid ; Yield 93%; $^1$H NMR (300 MHz, Chloroform-d) δ 9.18 (t, J = 2.1 Hz, 1H), 8.96 (d, J = 2.1 Hz, 2H), 6.40 (s, 1H), 3.54 (td, J = 7.3, 5.7 Hz, 2H), 1.77 - 1.63 (m, 2H), 1.50 – 1.16 (m, 18H), 0.89 (t, J = 7.3 Hz, 3H). $^{13}$C RMN (300 MHz, Chloroform-d) δ 162.76 (C7), 120.94 (C4), 138.21 (C1), 127.16 (C2 and C6), 148.64 (C3 and C5), 40.89 (C9), 31.90 (C10), 29.61 29.58 29.52 29.45 29.33 29.28 26.98 22.67 (C11-C19), 14.09 (C20). Infra-red (IR) - (n, cm-1) - 1720,43 (C=O). HRMS (ESI$^+$): *m/z* calculated for $C_{19}H_{29}N_3O_5$: 379.45078, found: 380.2153 (M+H$^+$). Purity by HPLC 98,5%.

Synthesis of *N*-tetradecyl-3,5-dinitrobenzamide **(14).** Following the described general procedure, 6 mmol (0,837 mL) of 3,5-dinitrobenzoyl chloride were dissolved in DCM (2,5 mL) and added to a solution in DCM (2,5 mL) of 9 mmol (2.371 mL) of n-tetradecylamine and 6 mmol (0,833 mL) of triethylamine. *N*-tetradecyl-3,5-dinitrobenzamide - yellow solid ; Yield 20%; $^1$H NMR (300 MHz, Chloroform-d) δ 9.17 (t, J = 2.0 Hz, 1H), 8.97 (d, J = 2.0 Hz, 2H), 6.49 (s, 1H), 3.53 (td, J = 7.3, 5.7 Hz, 2H), 1.81 - 1.58 (m, 4H), 1.48 - 1.18 (m, 20H), 0.89 (t, J = 7.3 Hz, 3H). $^{13}$C RMN (300 MHz, Chloroform-d) δ 162.70 (C7), 120.95 (C4), 138.23 (C1), 127.12 (C2 and C6), 148.67 (C3 and C5), 40.87 (C9), 31.91 (C10), 29.64 29.52 29.46 29.34 29.27 26.97 22.68 (C11-C21), 14.10 (C22). Infra-red (IR) - (n, cm-1) - 1720,43 (C=O). HRMS (ESI$^+$): *m/z* calculated for $C_{21}H_{33}N_3O_5$: 407.50394, found: 408.2451 (M+H$^+$). Purity by HPLC 98,4%.

Synthesis of *N*-hexadecyl-3,5-dinitrobenzamide **(15).** Following the described general procedure, 6 mmol (0,837 mL) of 3,5-dinitrobenzoyl chloride were dissolved in DCM (2,5 mL) and added to a solution in DCM (2,5 mL) of 9 mmol (2.670 mL) of n-hexadecylamine and 6 mmol (0,833 mL) of

triethylamine. *N*-hexadecyl-3,5-dinitrobenzamide - yellow solid ; Yield 86%; $^1$H NMR (300 MHz, Chloroform-d) δ 9.18 (t, J = 2.1 Hz, 1H), 8.95 (d, J = 2.1 Hz, 2H), 6.36 (s, 1H), 3.54 (td, J = 7.3, 5.7 Hz, 2H), 1.77 - 1.63 (m, 2H), 1.49 – 1.17 (m, 26H), 0.89 (t, J = 7.3 Hz, 3H). $^{13}$C RMN (300 MHz, Chloroform-d) δ 162.75 (C7), 120.95 (C4), 138.21 (C1), 127.14 (C2 and C6), 148.65 (C3 and C5), 40.89 (C9), 31.92 (C10), 29.68 29.52 29.45 29.35 29.28 26.98 22.68 (C11-C23), 14.10 (C24). Infra-red (IR) - (n, cm-1) - 1720,43 (C=O). HRMS (ESI$^+$): *m/z* calculated for $C_{23}H_{37}N_3O_5$: 435.5571, found: 436.2768 (M+H$^+$). Purity by HPLC 98,9%.

Synthesis of *N*-butyl-3-nitro-5-(trifluoromethyl)benzamide **(16).** Following the described general procedure, 6 mmol (0,967 mL) of 3-nitro-5-(trifluoromethyl)benzoyl chloride were dissolved in DCM (2,5 mL) and added to a solution in DCM (2,5 mL) of 9 mmol (0,885 mL) of n-butylamine and 6 mmol (0,833 mL) of triethylamine. *N*-butyl-3-nitro-5-(trifluoromethyl)benzamide - yellow solid ; Yield 84%; $^1$H NMR (300 MHz, Chloroform-d) δ 8.68 (br. s, 1H), 8.57 (br. s, 1H), 8.34 (br. s, 1H), 6.25 (s, 1H), 3.45 (td, J = 7.2, 5.7 Hz, 2H), 1.66 – 1.47 (m, 2H), 1.45 – 1.28 (m, 2H), 0.91 (t, J = 7.3 Hz, 3H). $^{13}$C RMN (300 MHz, Chloroform-d) δ 163.99 (C7), 120.78 (C4), 132.91 (q, $J_{CF}$ = 34.7 Hz, C5), 130.13 (C2), 125.03 (C6), 123.08 ($CF_3$), 148.45 (C3), 137.81 (C1), 40.57 (C9), 31.58 (C10), 20.25 (C11), 13.81 (C12). Infra-red (IR) - (n, cm-1) - 1720,43 (C=O). HRMS (ESI$^+$): *m/z* calculated for $C_{12}H_{13}F_3N_2O_3$: 290.2385096, found: 291.0952 (M+H$^+$). Purity by HPLC 99,7%.

Synthesis of *N*-hexyl-3-nitro-5-(trifluoromethyl)benzamide **(17).** Following the described general procedure, 6 mmol (0,967 mL) of 3-nitro-5-(trifluoromethyl)benzoyl chloride were dissolved in DCM (2,5 mL) and added to a solution in DCM (2,5 mL) of 9 mmol (1,182 mL) of n-hexylamine and 6 mmol (0,833 mL) of triethylamine. *N*-hexyl-3-nitro-5-(trifluoromethyl)benzamide - white solid ; Yield 30%; $^1$H NMR (300 MHz, Chloroform-d) δ 8.76 (br. s, 1H), 8.63 (br. s, 1H), 8.42 (br. s, 1H), 6.25 (s, 1H), 3.53 (td, J = 7.3, 5.7 Hz, 2H), 1.75 – 1.62 (m, 2H), 1.48 – 1.23 (m, 6H), 0.93 (t, J = 7.3 Hz, 3H). $^{13}$C RMN (300 MHz, Chloroform-d) δ 163,68 (C7), 120.65 (C4), 132.87 (q, $J_{CF}$ = 34.6 Hz, C5), 129,95 (C2), 124,73 (C6), 122,95 ($CF_3$), 148.36 (C3), 137.69 (C1), 40.74 (C9), 31.43 (C10), 29.42 26.76 22.51 (C11-C13), 13.95 (C14). Infra-red (IR) - (n, cm-1) - 1720,43 (C=O). HRMS (ESI$^+$): *m/z* calculated for $C_{14}H_{17}F_3N_2O_3$: 318.2916696, found: 319.1192 (M+H$^+$). Purity by HPLC 99,6%.

Synthesis of *N*-decyl-3-nitro-5-(trifluoromethyl)benzamide **(19).** Following the described general procedure, 6 mmol (0,967 mL) of 3-nitro-5-(trifluoromethyl)benzoyl chloride were dissolved in DCM (2,5 mL) and added to a solution in DCM (2,5 mL) of 9 mmol (1,776 mL) of n-decylamine and 6 mmol (0,833 mL) of triethylamine. *N*-decyl-3-nitro-5-(trifluoromethyl)benzamide - white solid ; Yield 30%; $^1$H NMR (300 MHz, Chloroform-d) δ 8.77 (br. s, 1H), 8.63 (br. s, 1H), 8.42 (br. s, 1H), 6.30 (s, 1H), 3.52 (td, J = 7.3, 5.7 Hz, 2H), 1.75 - 1.60 (m, 2H), 1.49 – 1.17 (m, 14H), 0.89 (t, J = 7.3 Hz, 3H). $^{13}$C RMN (300 MHz, Chloroform-d) δ 163.73 (C7), 148.55 (C3), 137.87 (C1), 133.34 (C5), 132.89 (C2), 130.06, 124.79 (C6), 123.13 ($CF_3$), 120.81, 40.88 (C9), 32.01 (C10), 29.67 29.64 29.42 27.11 22.80 (C11-C17), 14.22 (C18). Infra-red (IR) - (n, cm-1) - 1720,43 (C=O). HRMS (ESI$^+$): *m/z* calculated for $C_{18}H_{25}F_3N_2O_3$: 388.4245696, found: 375.1896 (M+H$^+$). Purity by HPLC 98,7%.

Synthesis of *N*-dodecyl-3-nitro-5-(trifluoromethyl)benzamide **(20).** Following the described general procedure, 6 mmol (0,967 mL) of 3-nitro-5-(trifluoromethyl)benzoyl chloride were dissolved in DCM (2,5 mL) and added to a solution in DCM (2,5 mL) of 9 mmol (2.074 mL) of n-dodecylamine and 6 mmol (0,833 mL) of triethylamine. *N*-dodecyl-3-nitro-5-(trifluoromethyl)benzamide - white solid ; Yield 45%; $^1$H NMR (300 MHz, Chloroform-d) δ 8.76 (br. s, 1H), 8.63 (br. s, 1H), 8.42 (br. s, 1H), 6.29 (s, 1H), 3.52 (td, J = 7.3, 5.7 Hz, 2H), 1.76 – 1.60 (m, 2H), 1.49 – 1.18 (m, 18H), 0.89 (t, J = 7.3 Hz, 3H). $^{13}$C RMN (300 MHz, Chloroform-d) δ 163.75 (C7), 148.54 (C3), 137.86 (C1), 133.32 (C5), 132.86 (C2), 130.12, 124.82 (C6), 124.43, 123.12 ($CF_3$), 120.81, 40.89 (C9), 32.05 (C10), 29.77 29.72 29.64, 29.47 29.42 27.12 26.36 25.25 22.82 (C11-C19), 14.23 (C20). Infra-red (IR) - (n, cm-1) - 1720,43 (C=O). HRMS (ESI$^+$): *m/z* calculated for $C_{20}H_{29}F_3N_2O_3$: 402.4511496, found: 403.2173 (M+H$^+$). Purity by HPLC 99,6%.

**$^1$H and $^{13}$C NMR spectra of the compounds.**

*N*-butyl-benzamide (**1**) – $^{1}$H and $^{13}$C NMR spectra

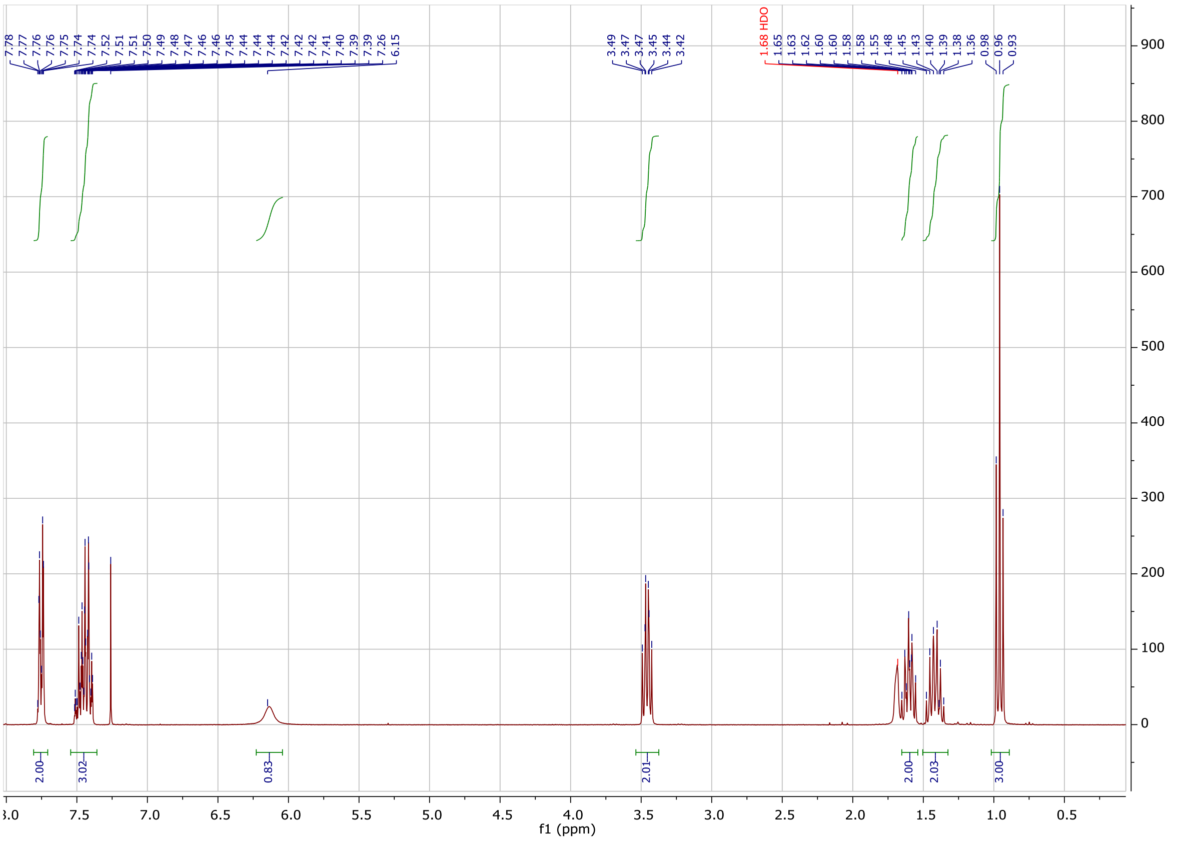


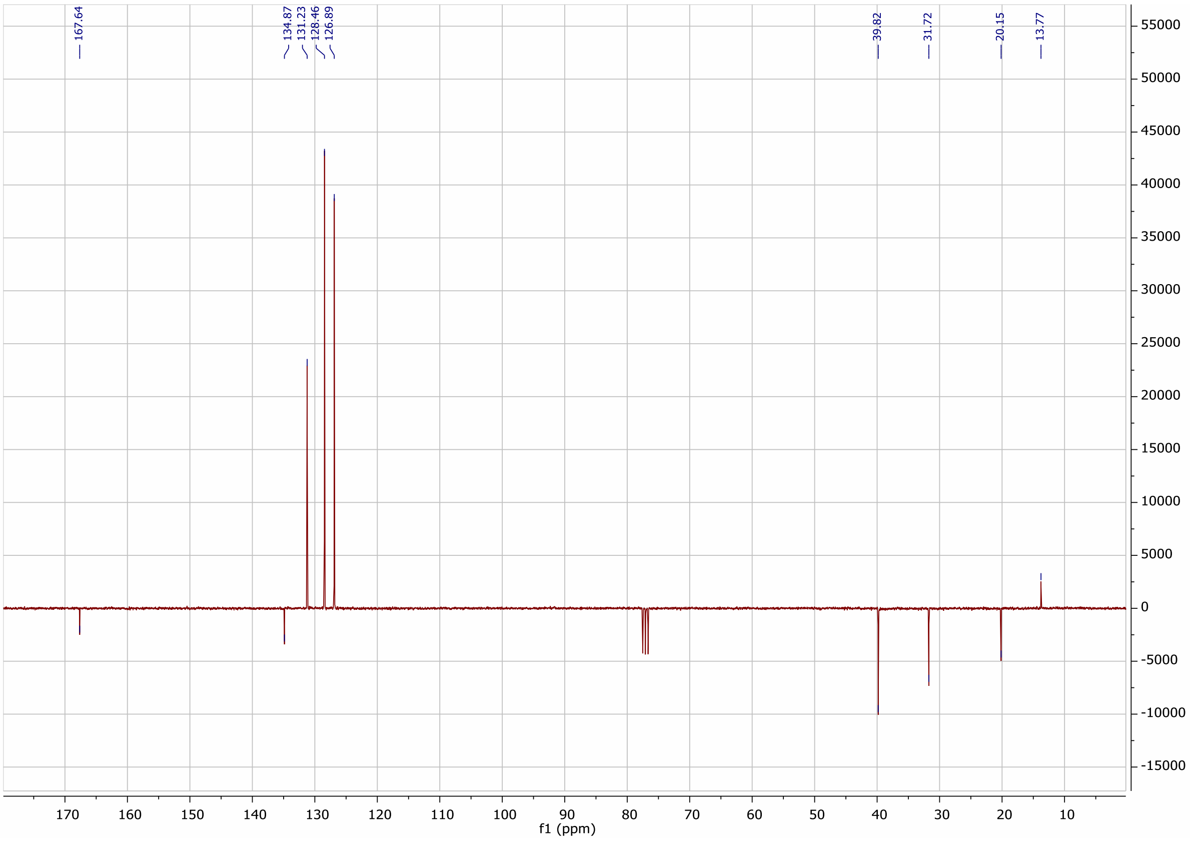


*N*-hexyl-benzamide (**2**) – $^{1}$H and $^{13}$C NMR spectra

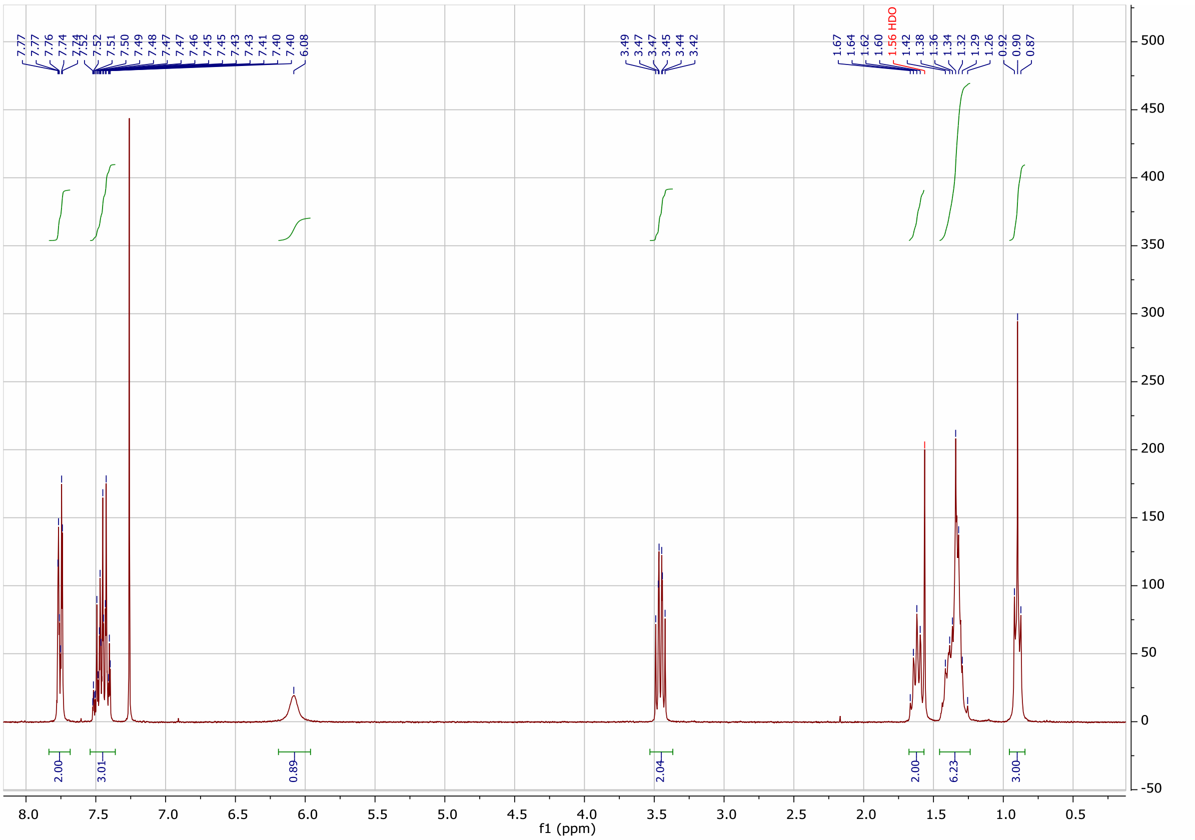


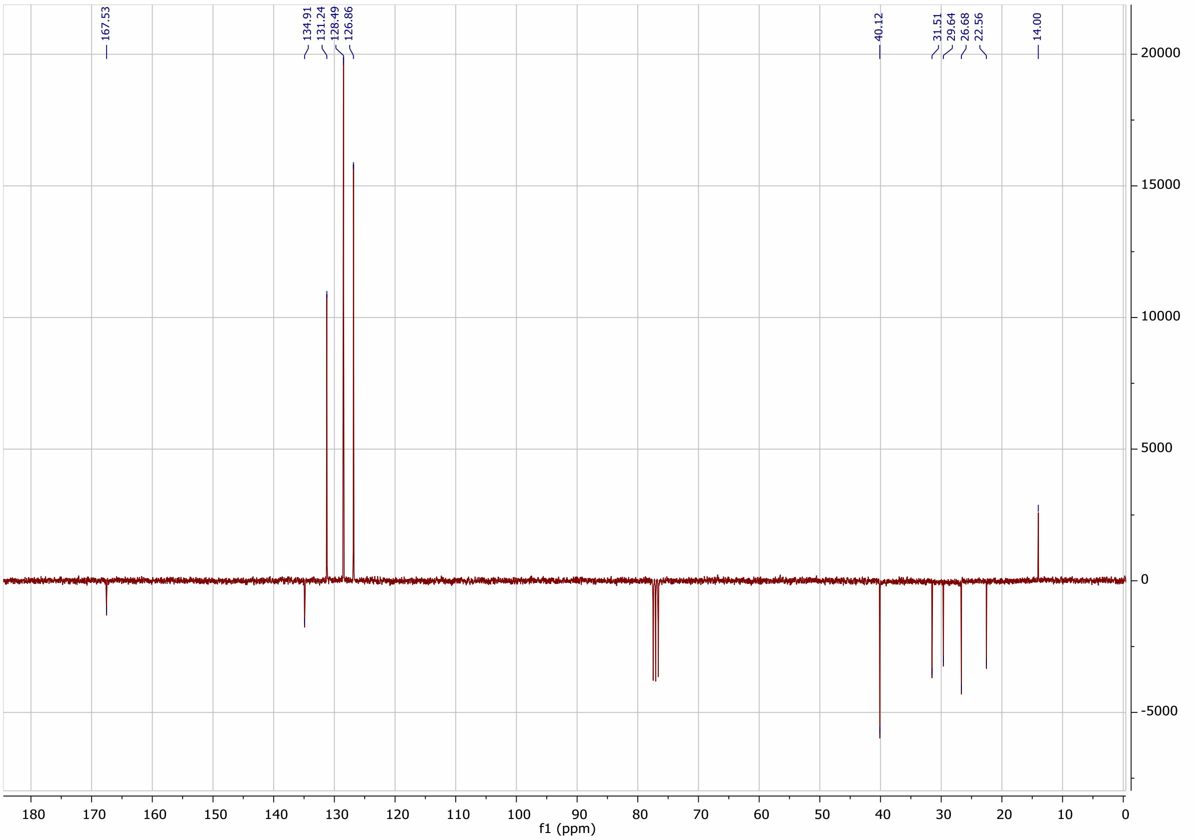


*N*-octyl-benzamide (**3**) – $^{1}$H and $^{13}$C NMR spectra

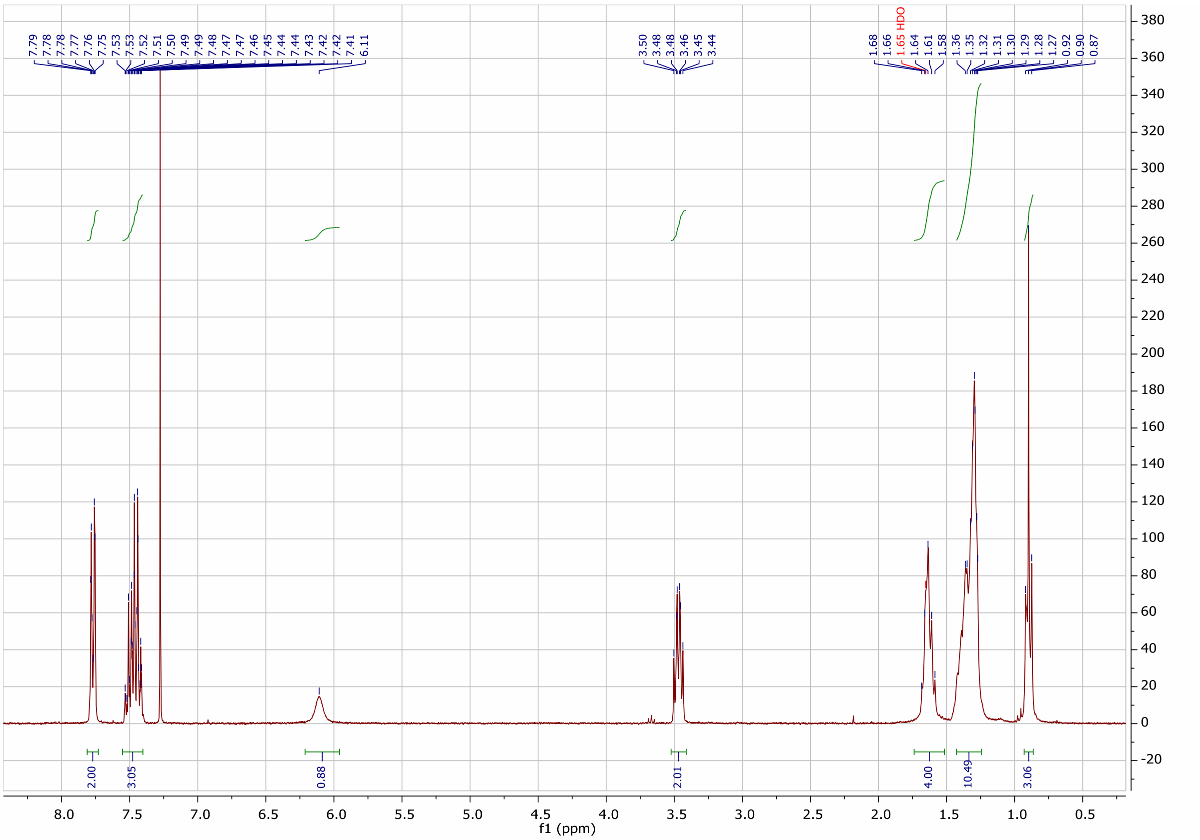


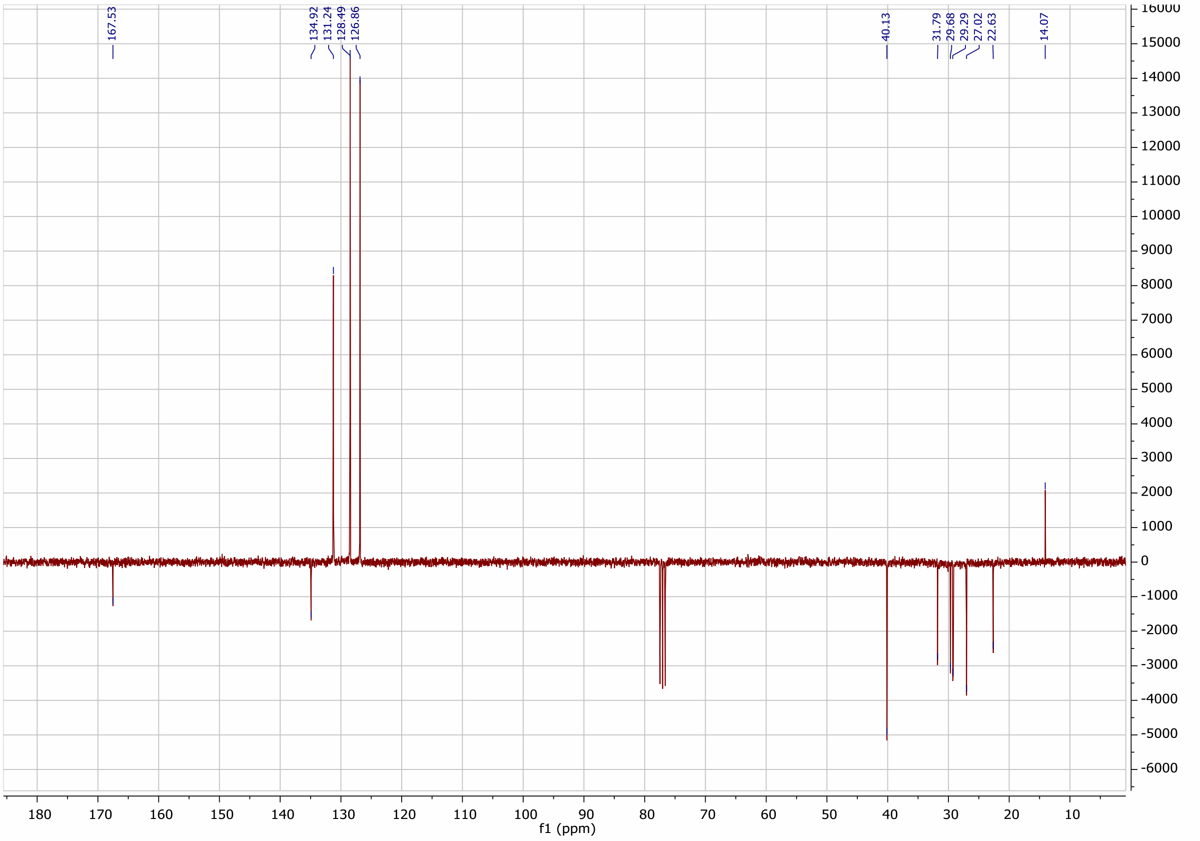


*N*-dodecyl-benzamide (**4**) – $^{1}$H and $^{13}$C NMR spectra

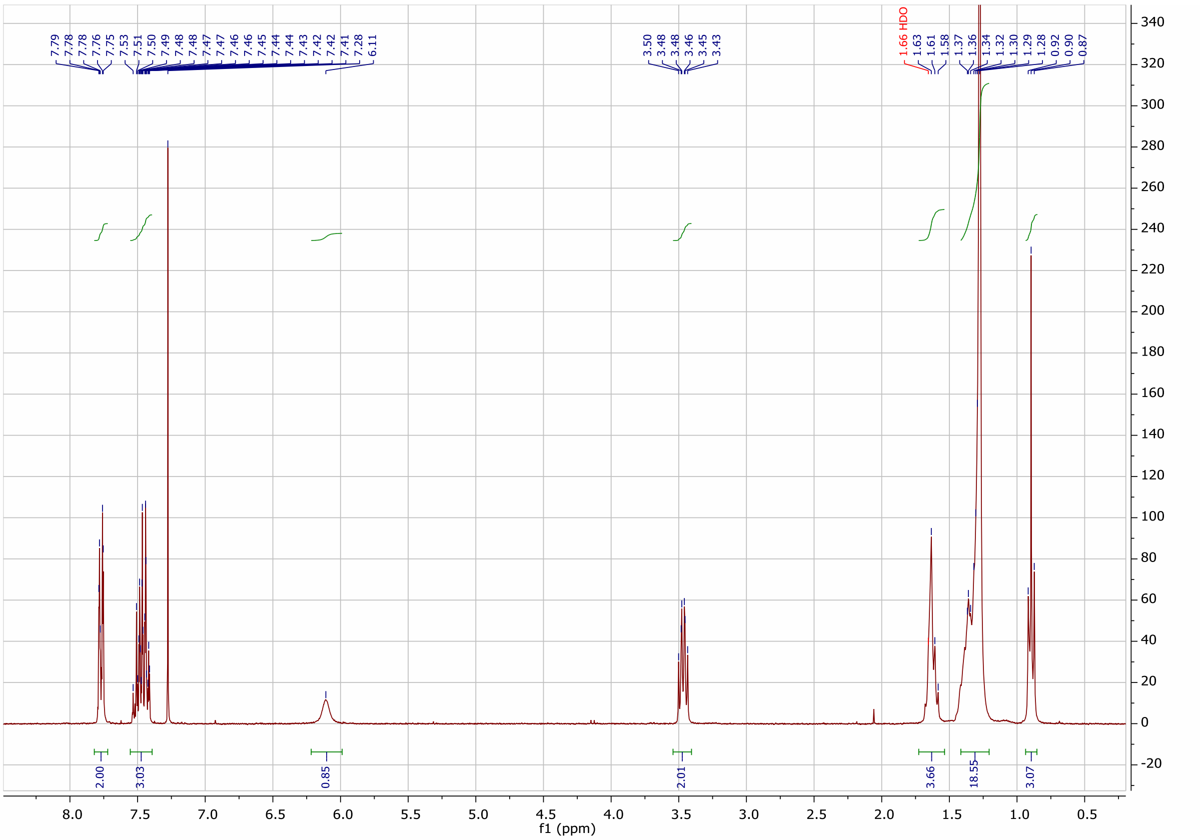

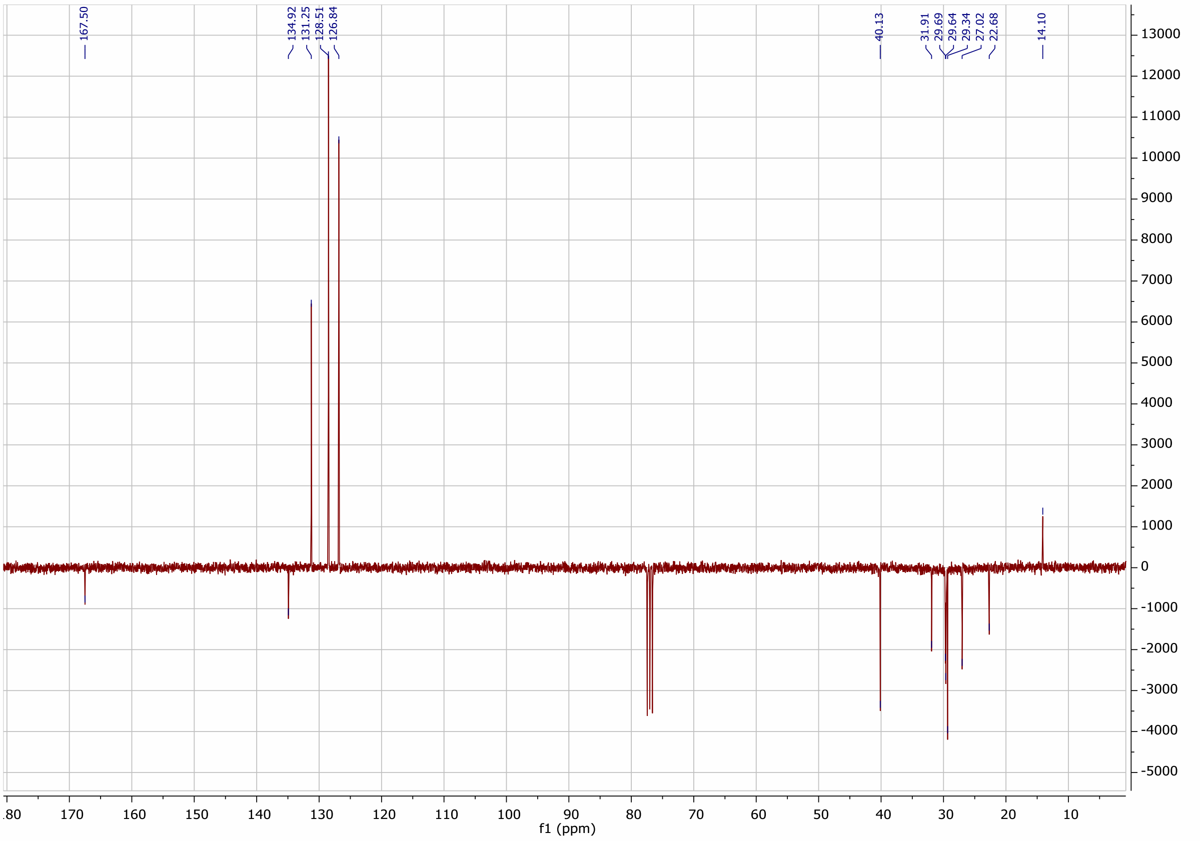


*N*-butyl-4-nitrobenzamide (**5**) – $^{1}$H and $^{13}$C NMR spectra

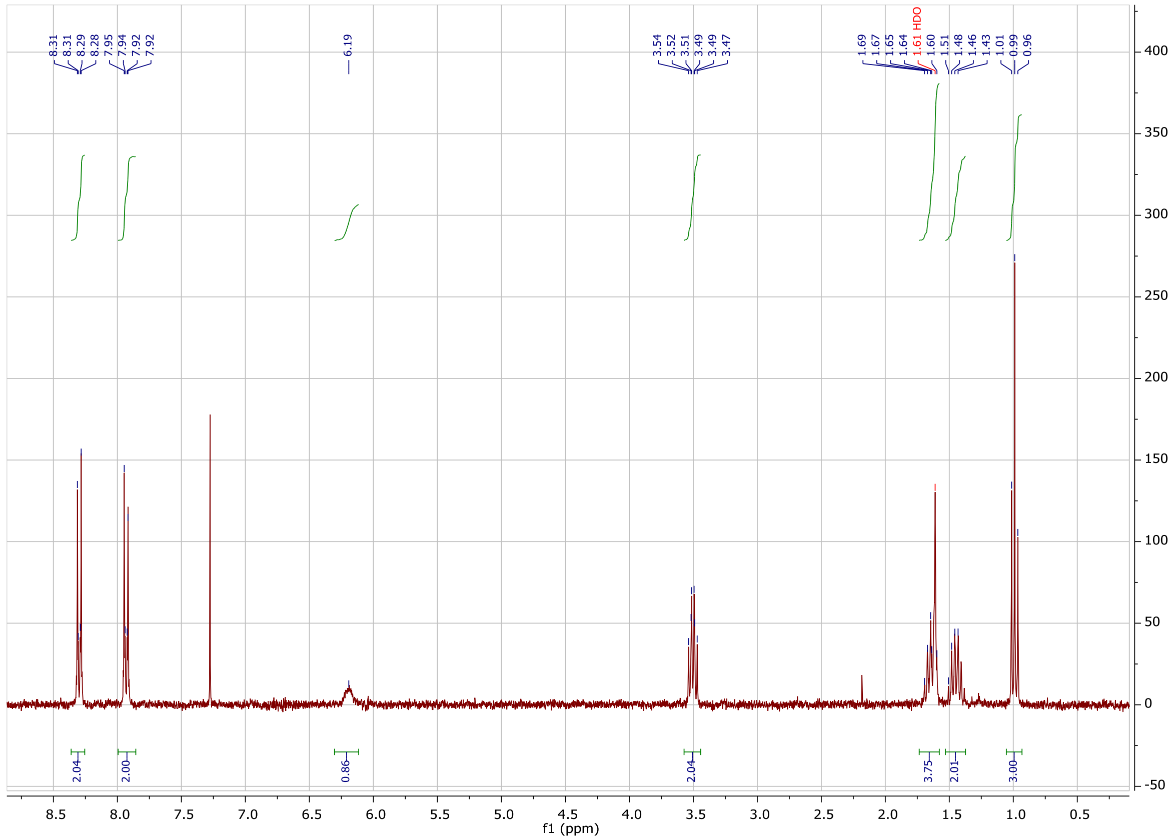

8.31
8.31
8.29
8.28
7.95
7.94
7.92
7.92
6.19
3.54
3.52
3.51
3.49
3.49
3.47
1.69
1.67
1.65
1.64
1.61 HDO
1.60
1.51
1.48
1.46
1.43
1.01
0.99
0.96
2.04
2.00
0.86
2.04
3.75
2.01
3.00
f1 (ppm)


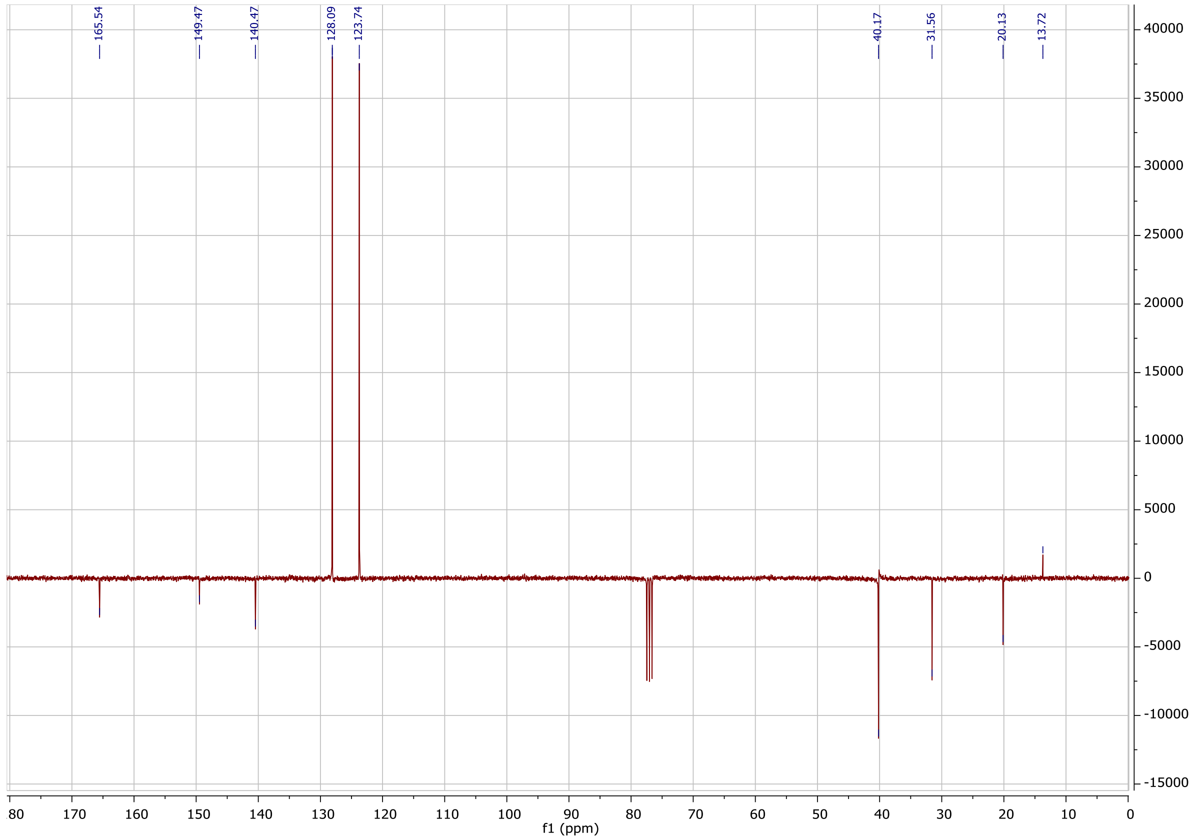

165.54
149.47
140.47
128.09
123.74
40.17
31.56
20.13
13.72
f1 (ppm)

*N*-hexyl-4-nitrobenzamide (**6**) – $^{1}$H and $^{13}$C NMR spectra

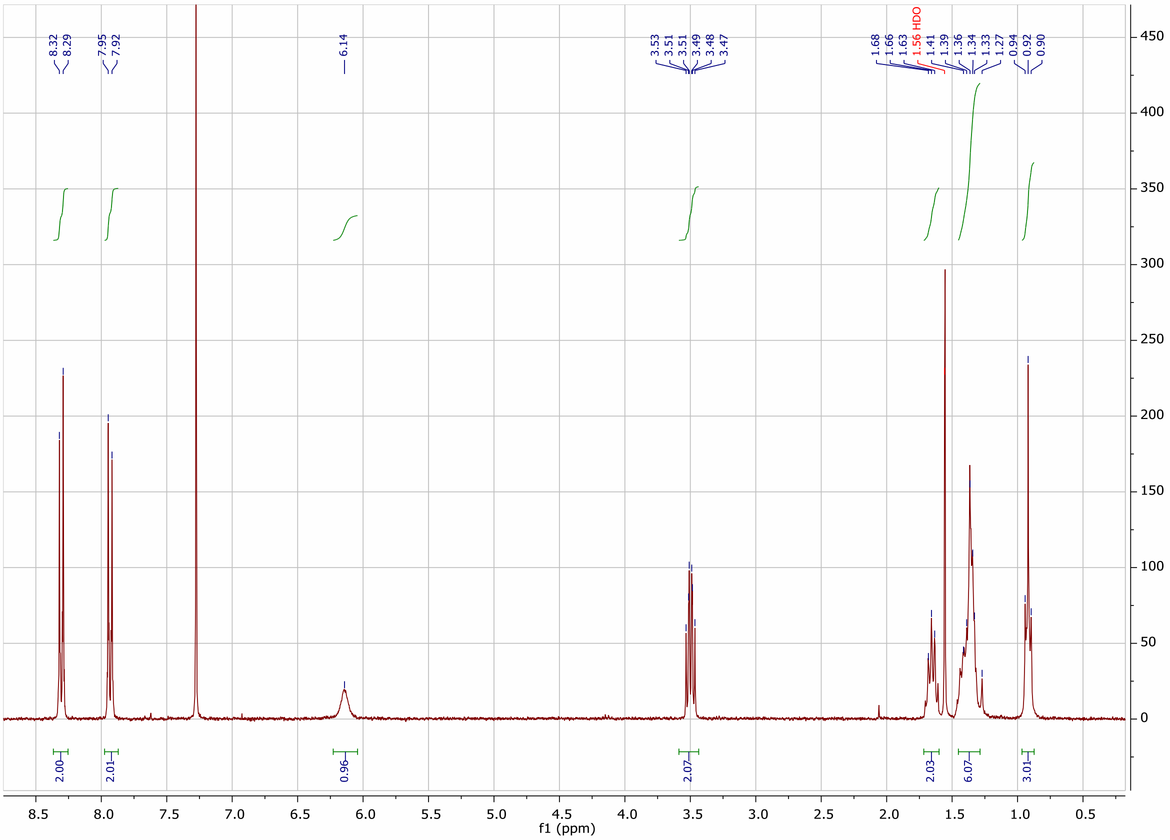


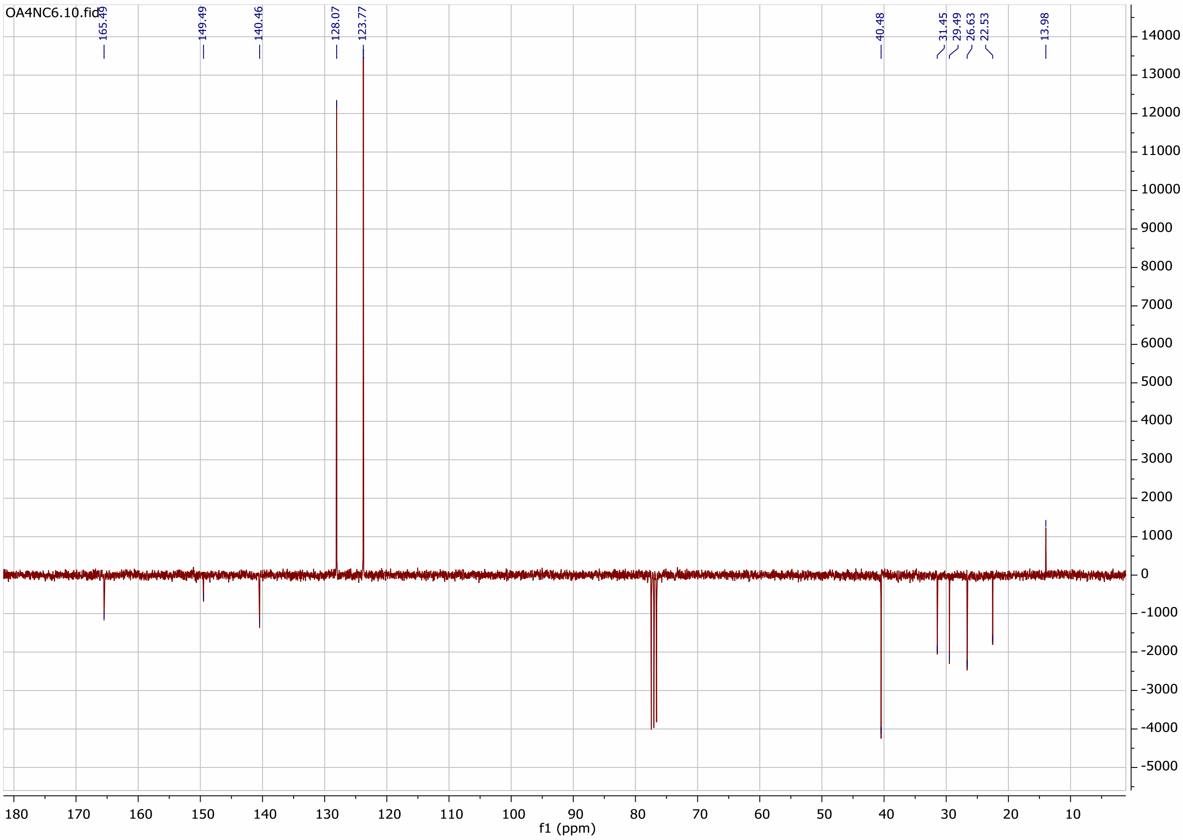

*N*-octyl-4-nitrobenzamide (**7**) - $^{1}$H and $^{13}$C NMR spectra

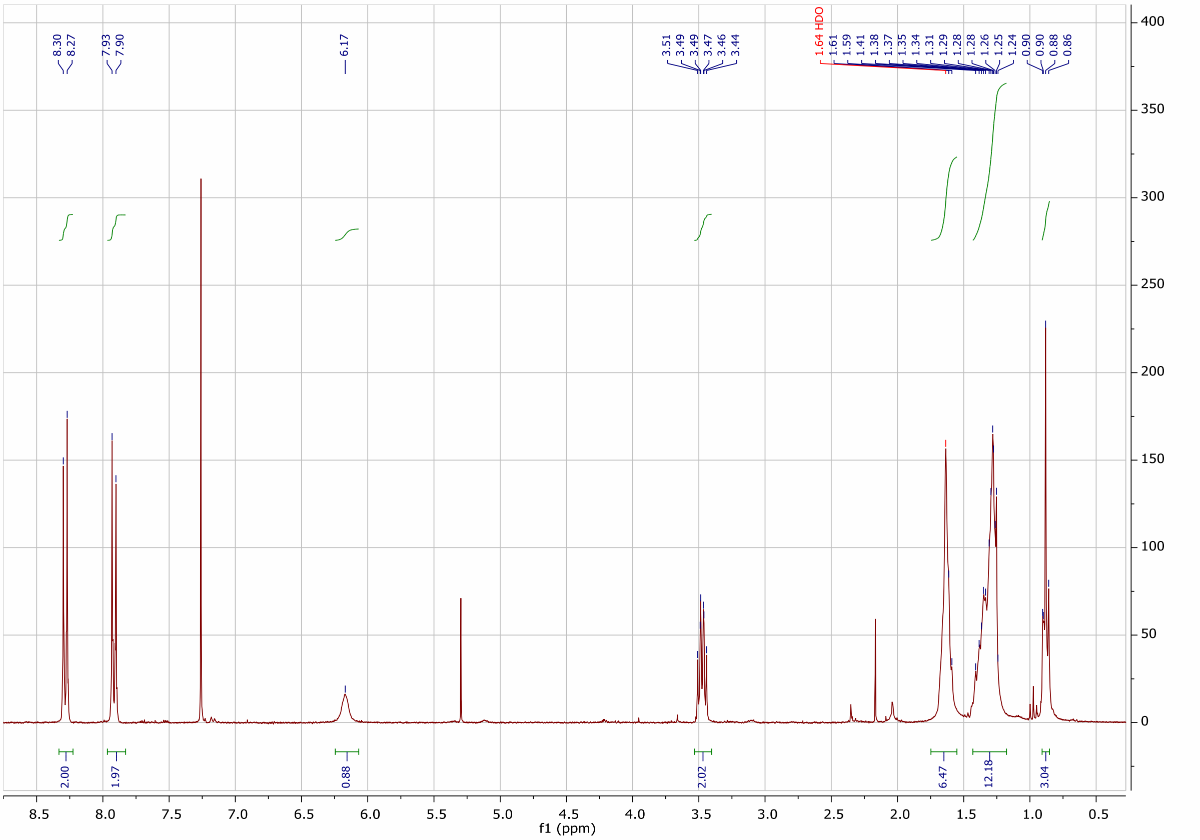


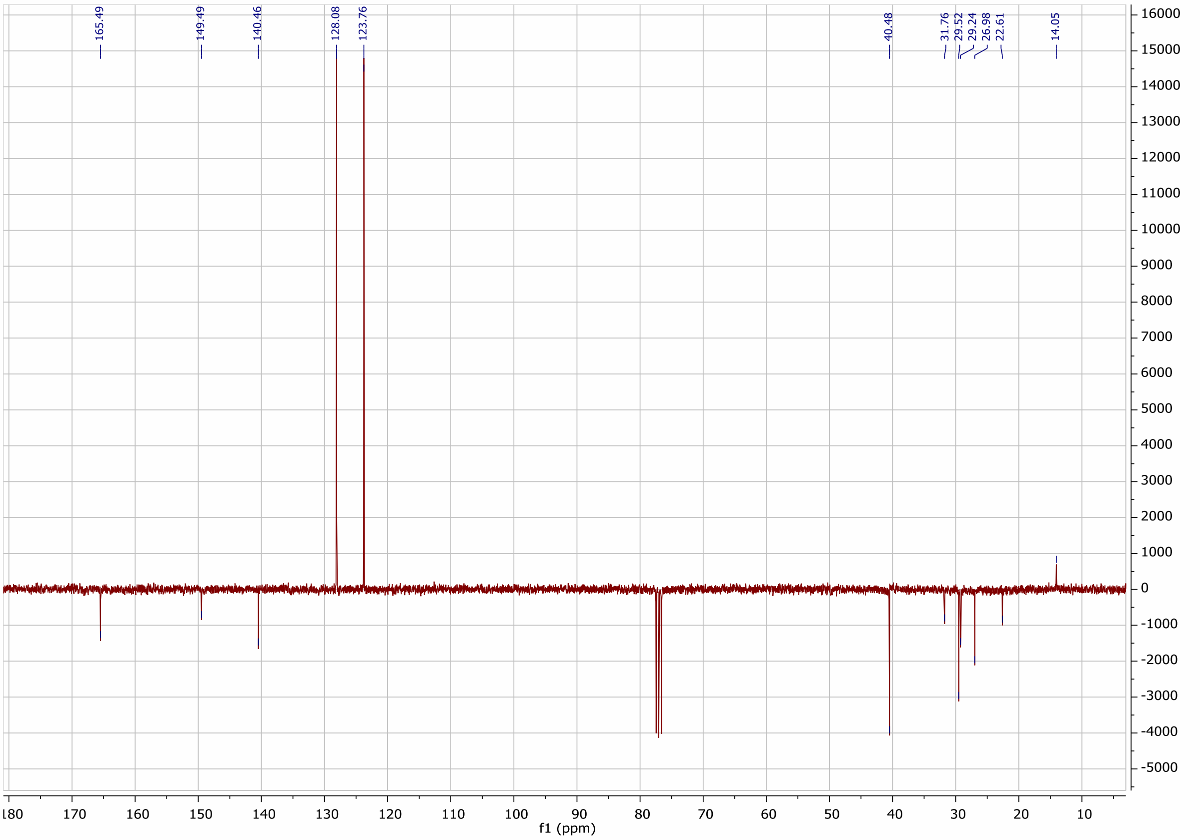


*N*-dodecyl-4-nitrobenzamide (**8**) - $^{1}$H NMR spectra

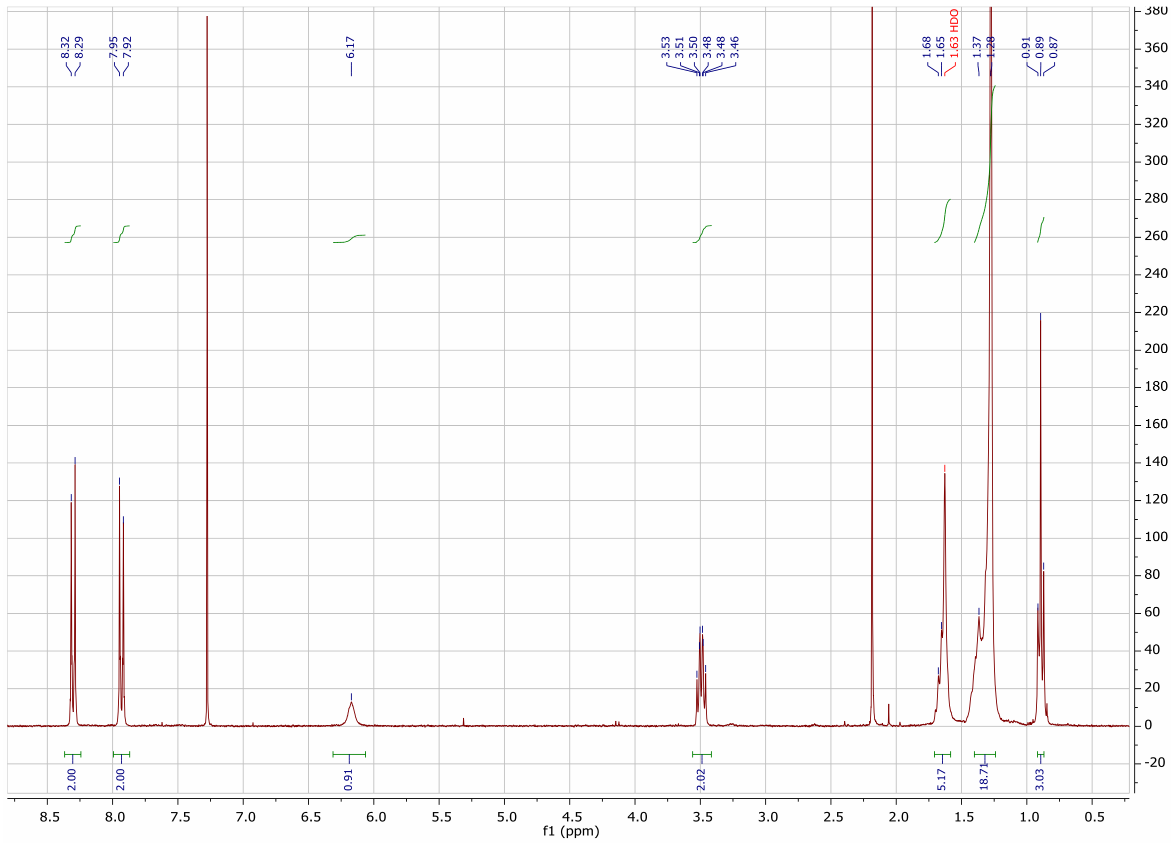

*N*-butyl-3,5-dinitrobenzamide (**9**) - $^{1}$H and $^{13}$C NMR spectra

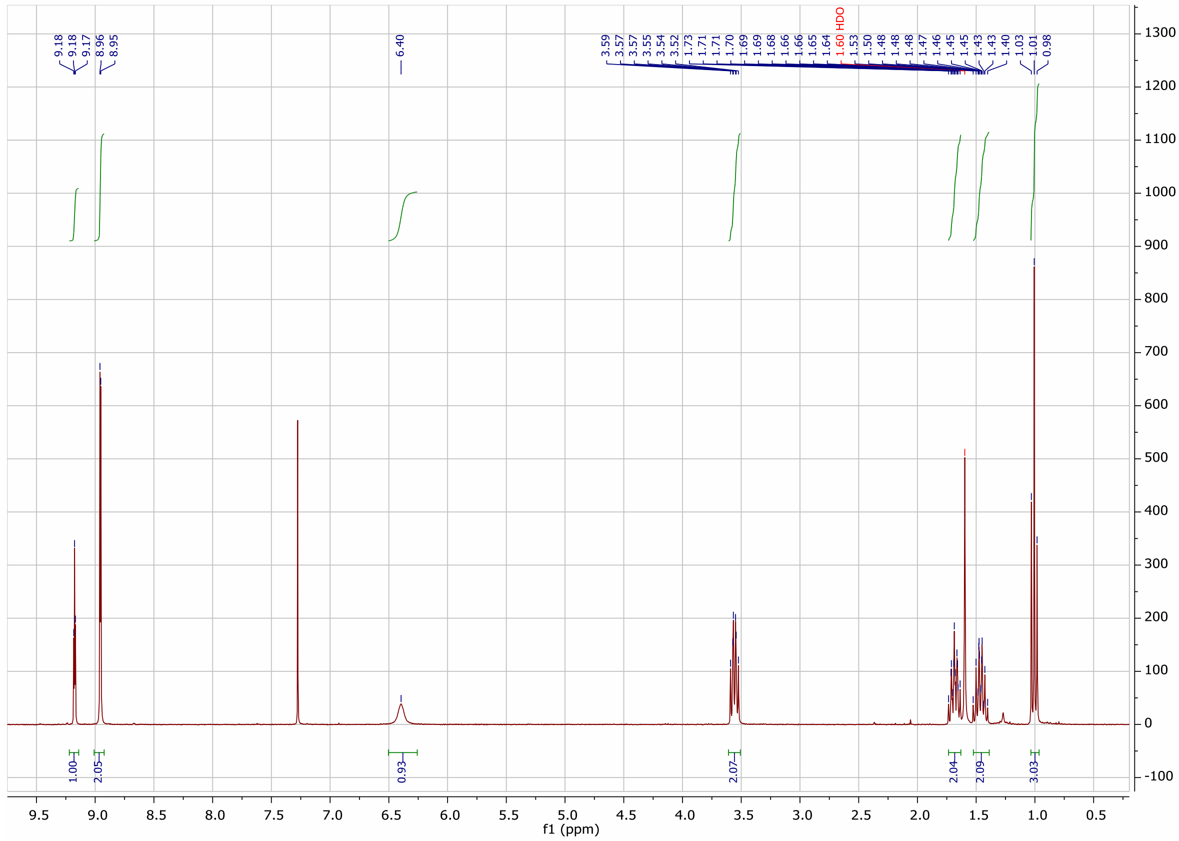

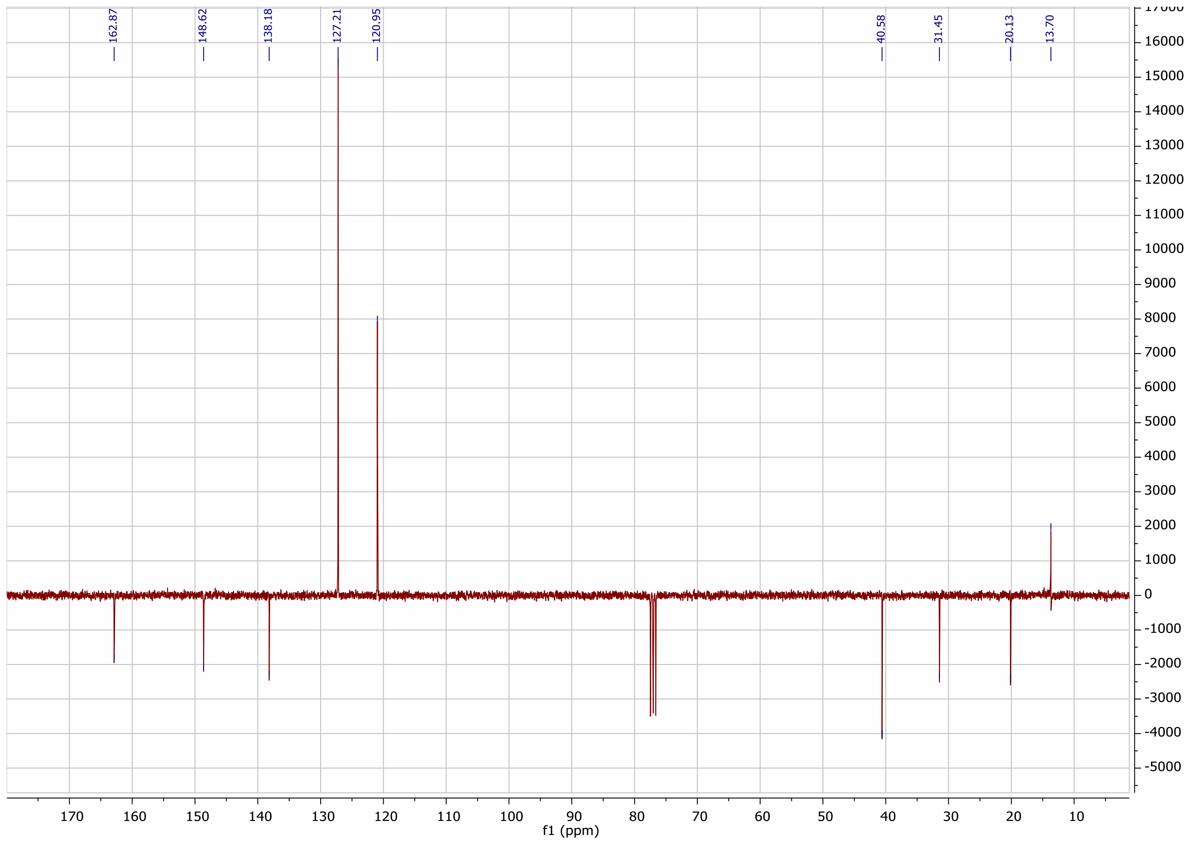

*N*-hexyl-3,5-dinitrobenzamide (**10**) - $^{1}$H and $^{13}$C NMR spectra

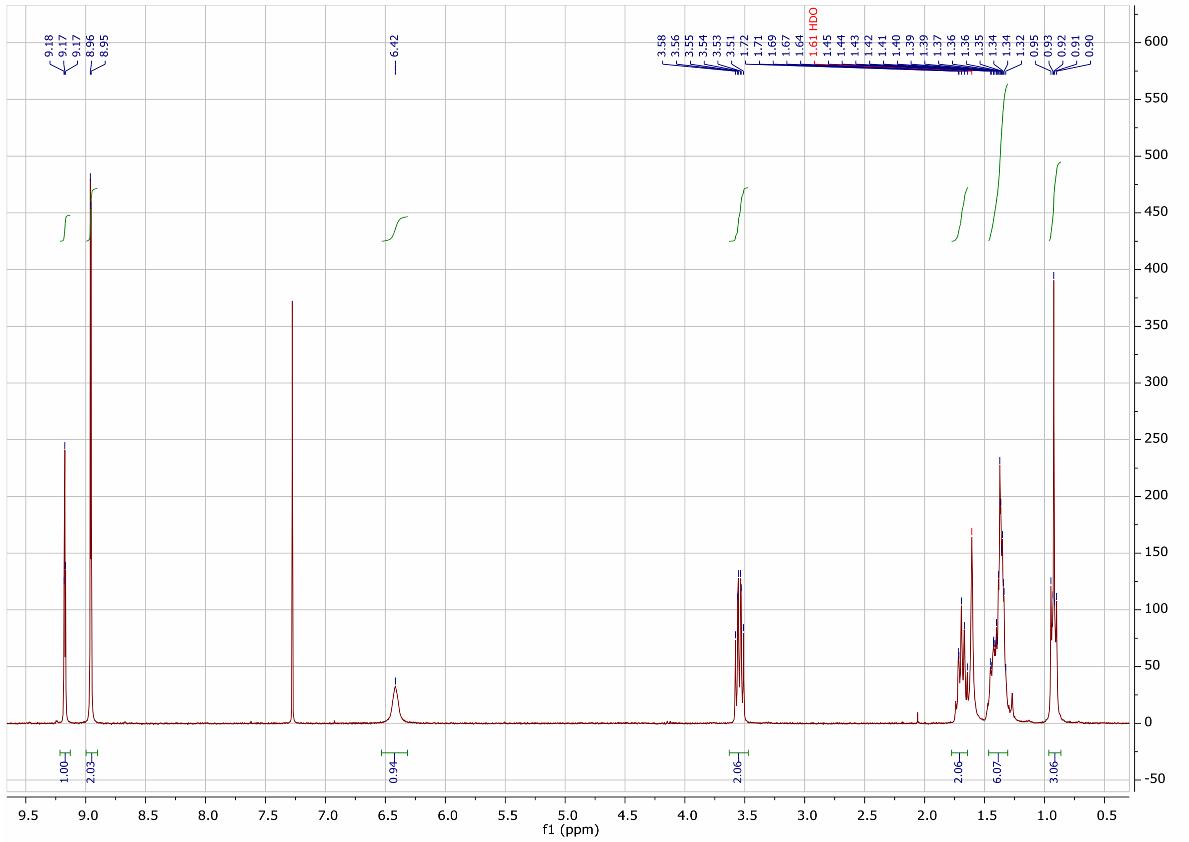

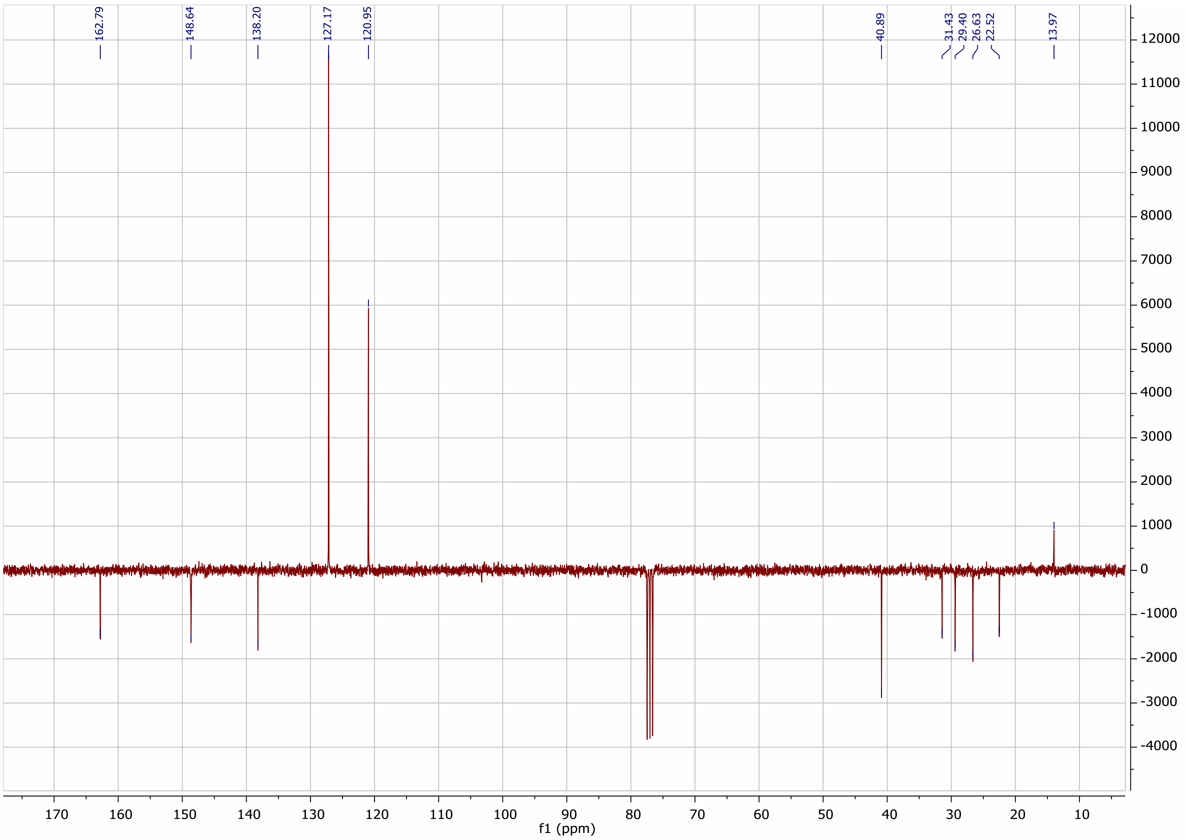


*N*-octyl-3,5-dinitrobenzamide (**11**) - $^{1}$H and $^{13}$C NMR spectra

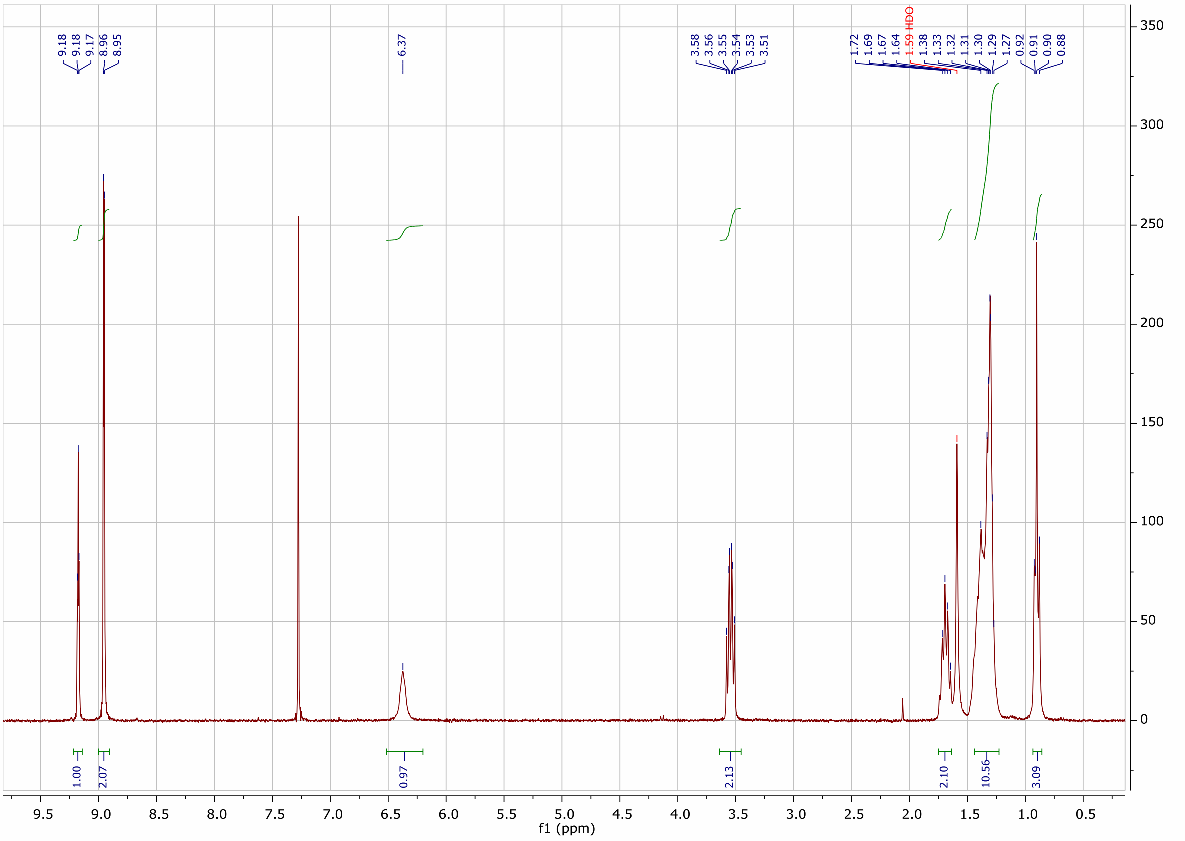

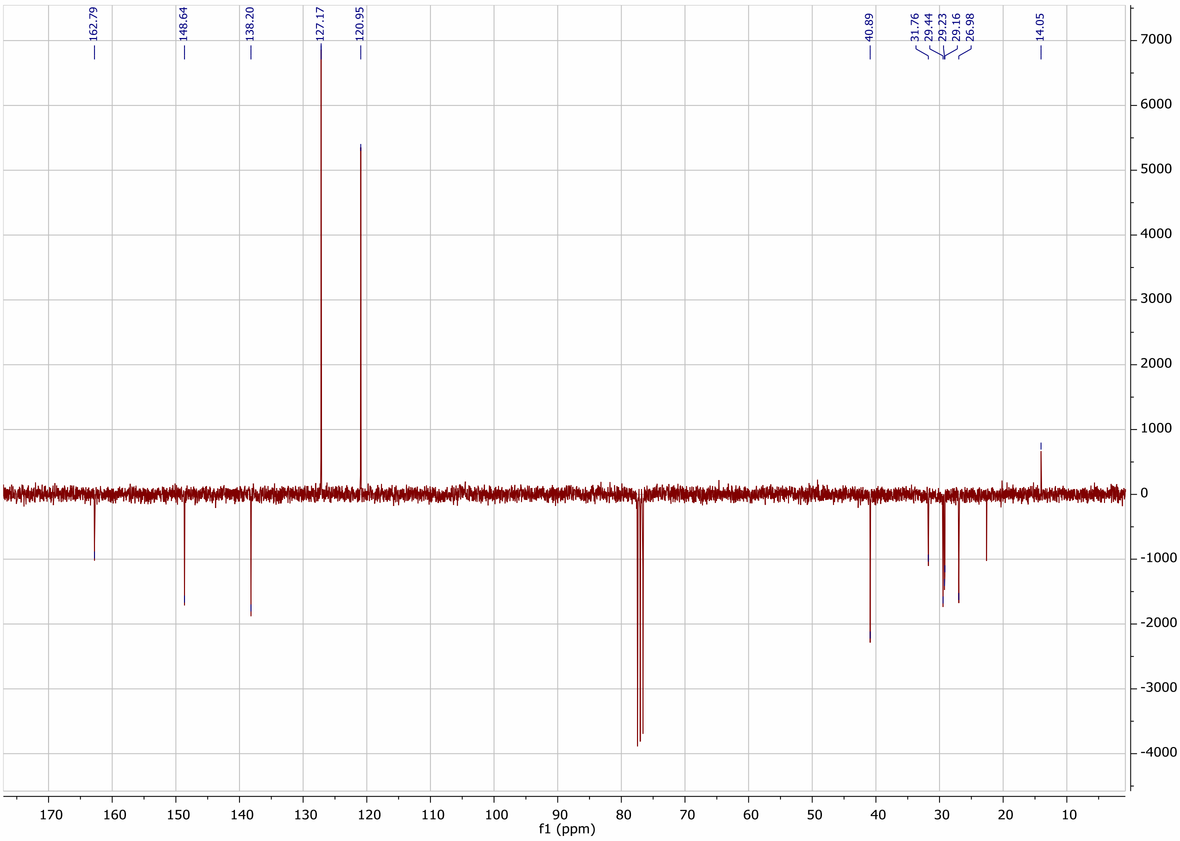


*N*-decyl-3,5-dinitrobenzamide **(12)** - $^{1}$H and $^{13}$C NMR spectra

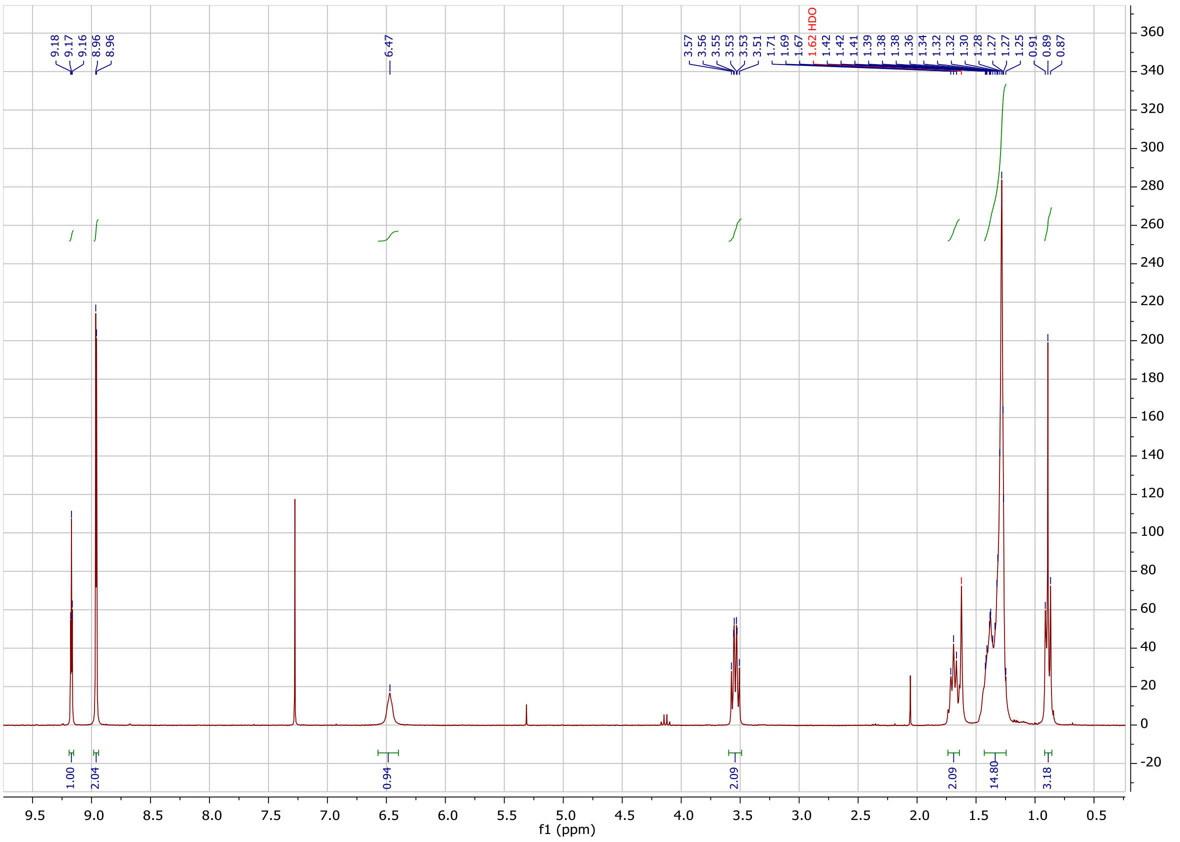

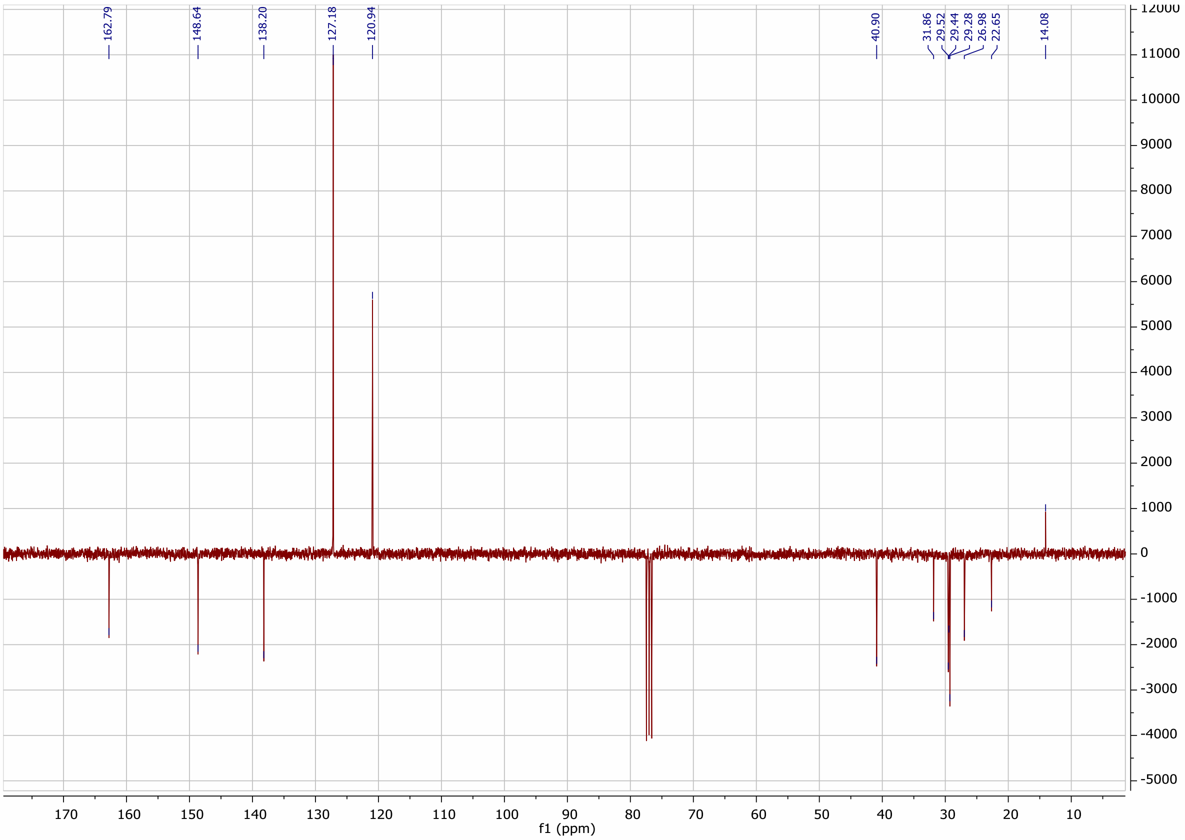


*N*-dodecyl-3,5-dinitrobenzamide **(13)** - $^{1}$H and $^{13}$C NMR spectra

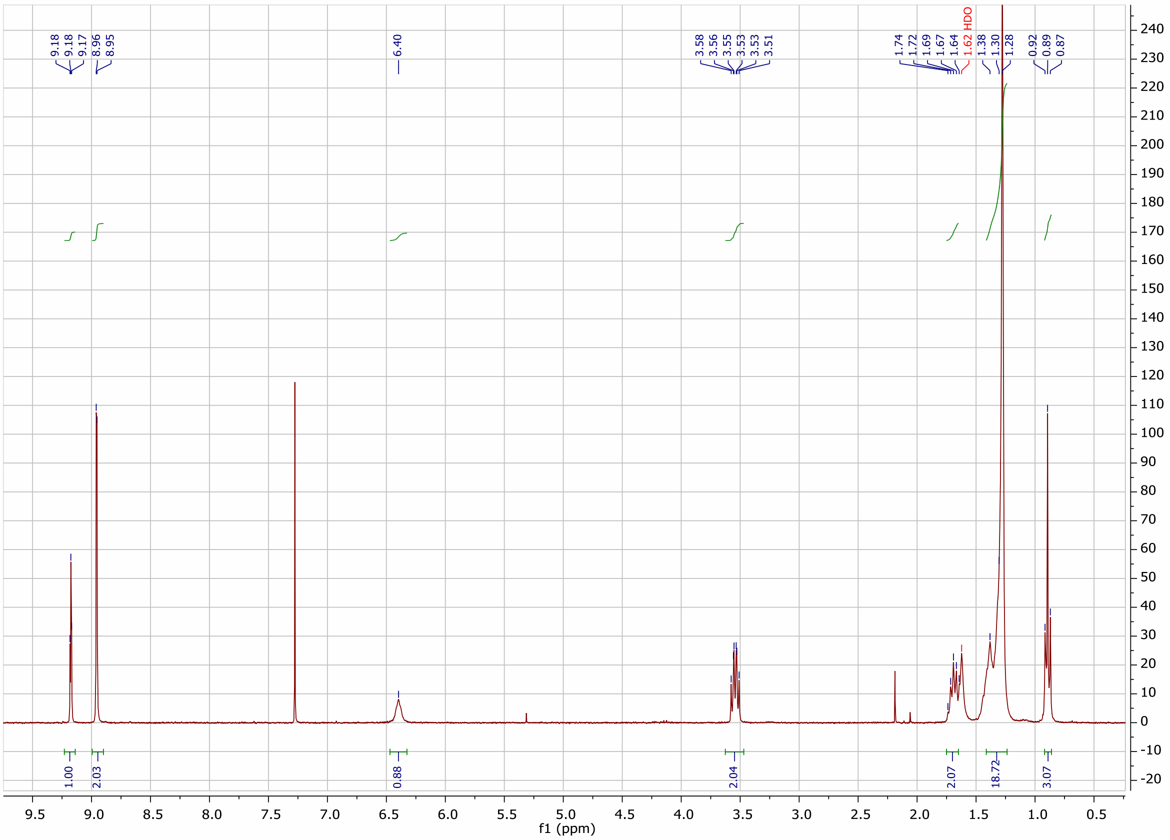

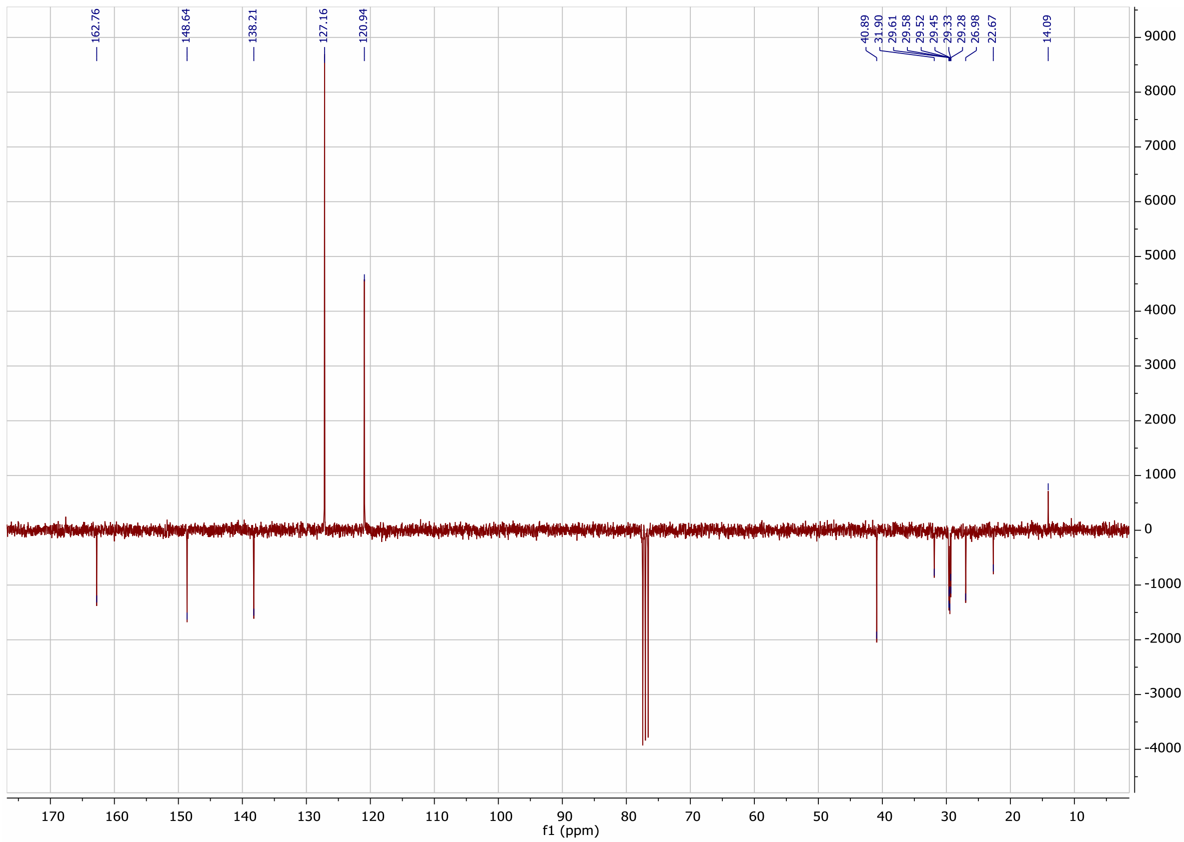


*N*-tetradecyl-3,5-dinitrobenzamide **(14)** - $^{1}H$ and $^{13}C$ NMR spectra

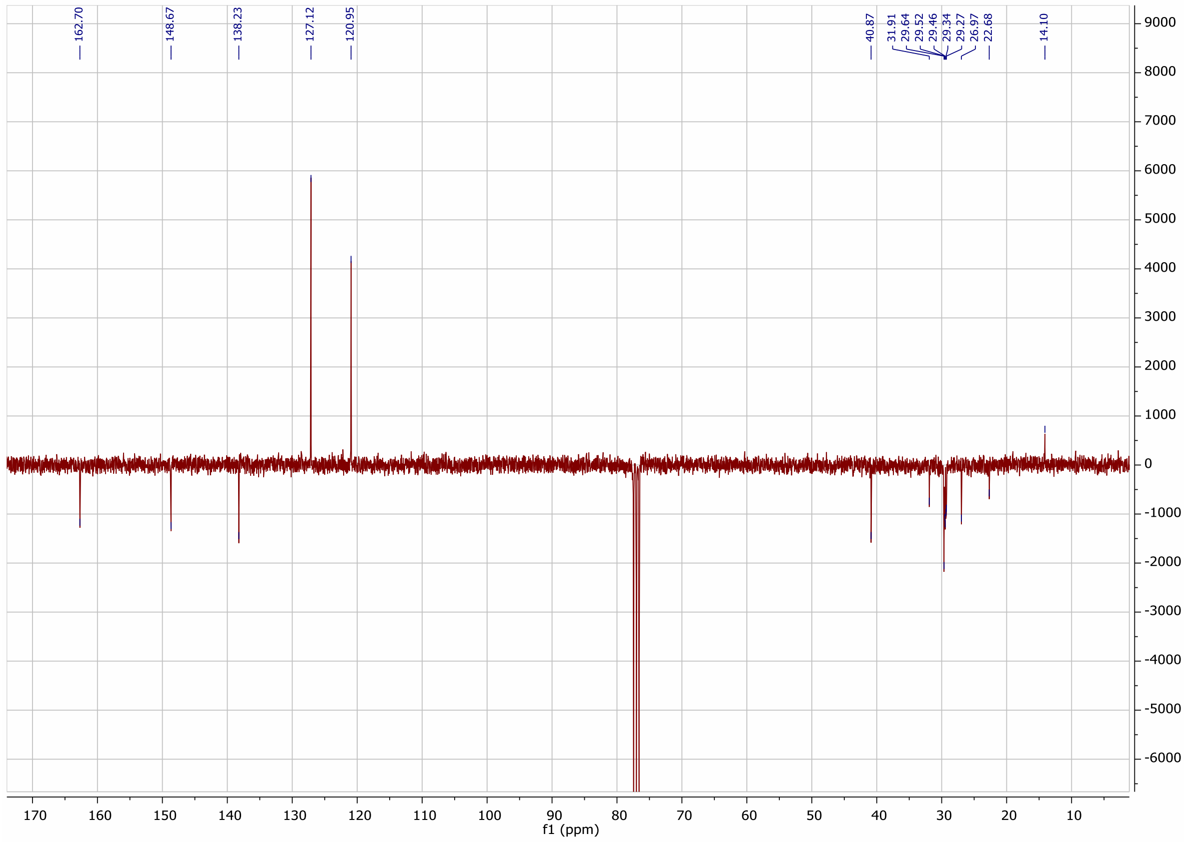

*N*-hexadecyl-3,5-dinitrobenzamide **(15)** - $^{1}$H and $^{13}$C NMR spectra

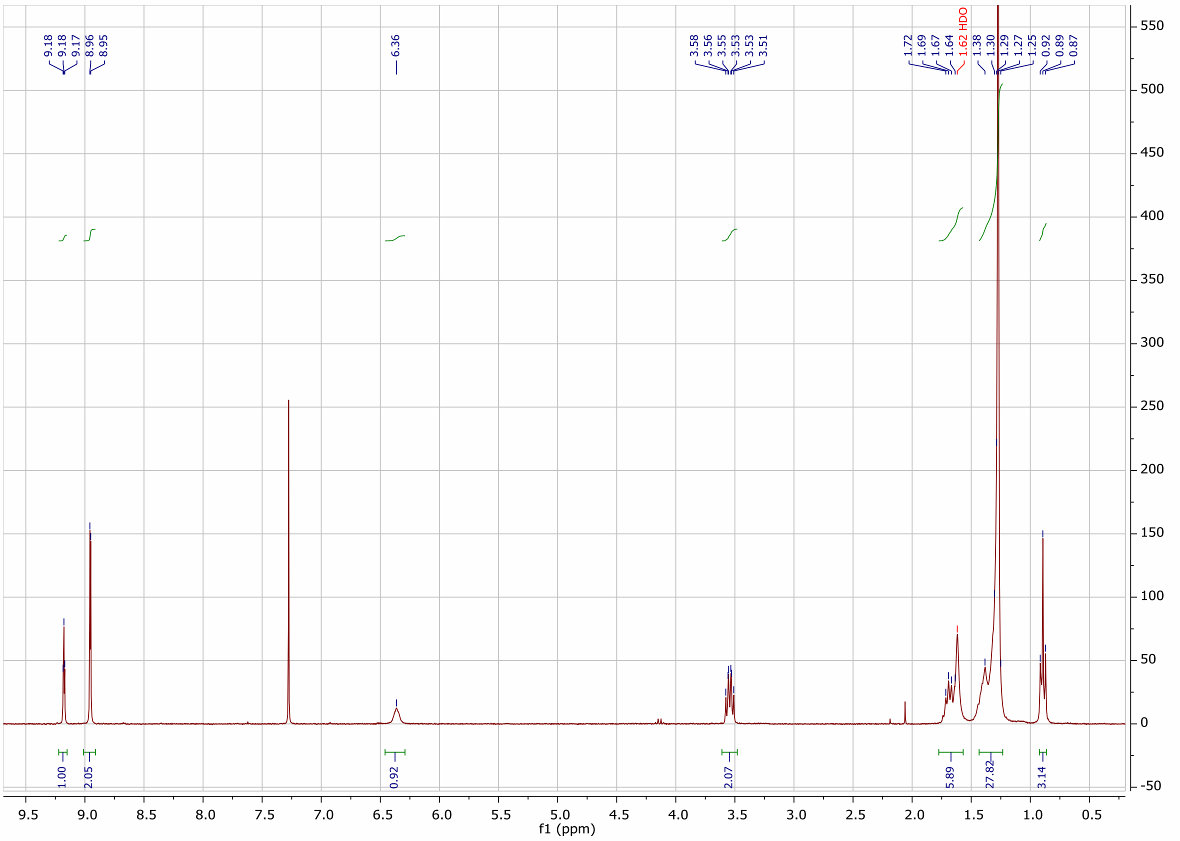

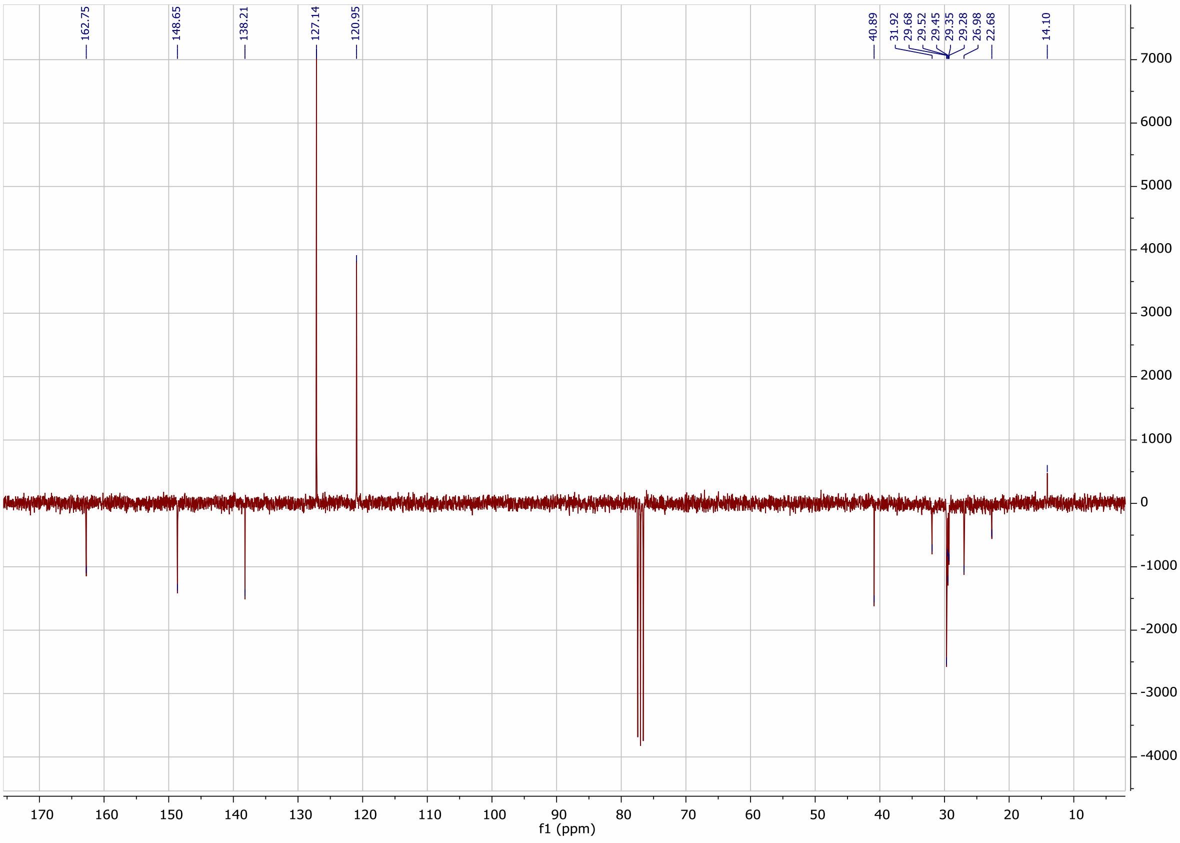

*N*-butyl-3-nitro-5-(trifluoromethyl)benzamide **(16)** - $^{1}$H and $^{13}$C NMR spectra

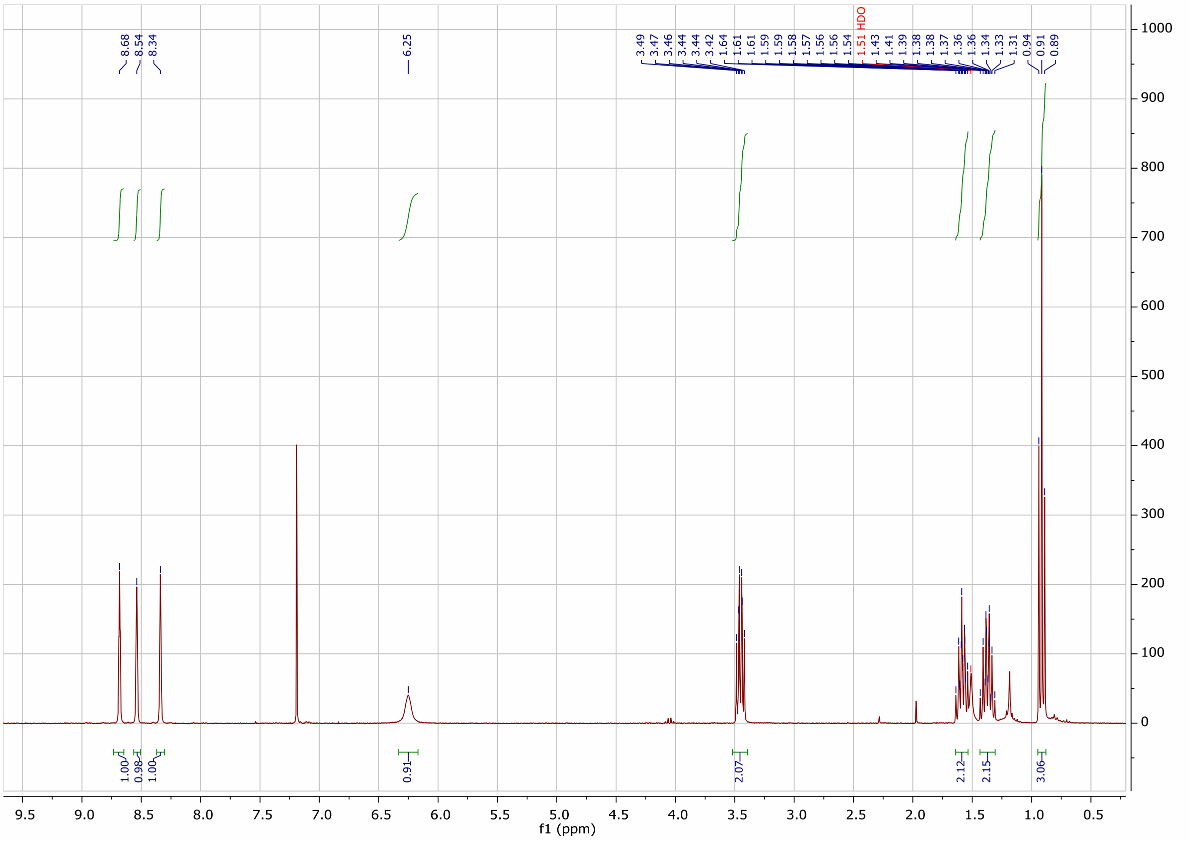


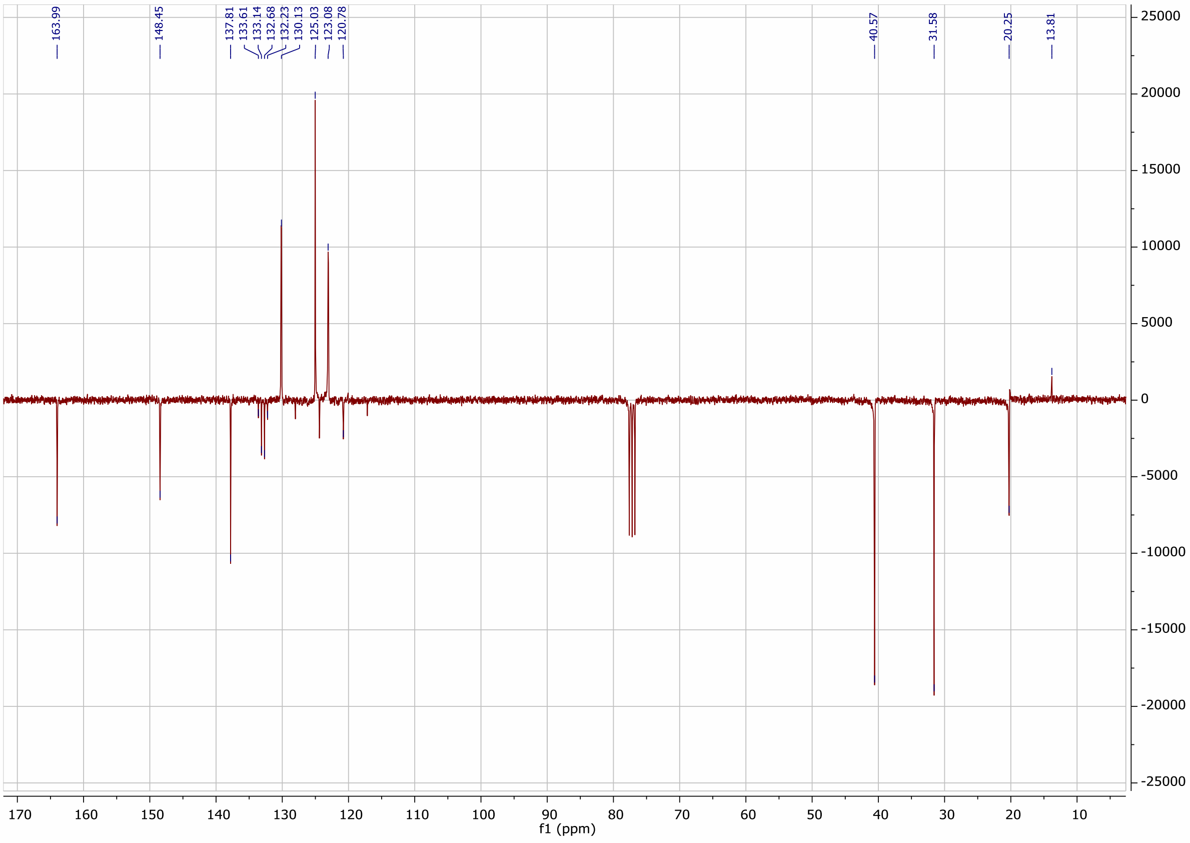

*N*-hexyl-3-nitro-5-(trifluoromethyl)benzamide **(17)** - $^{1}$H and $^{13}$C NMR spectra

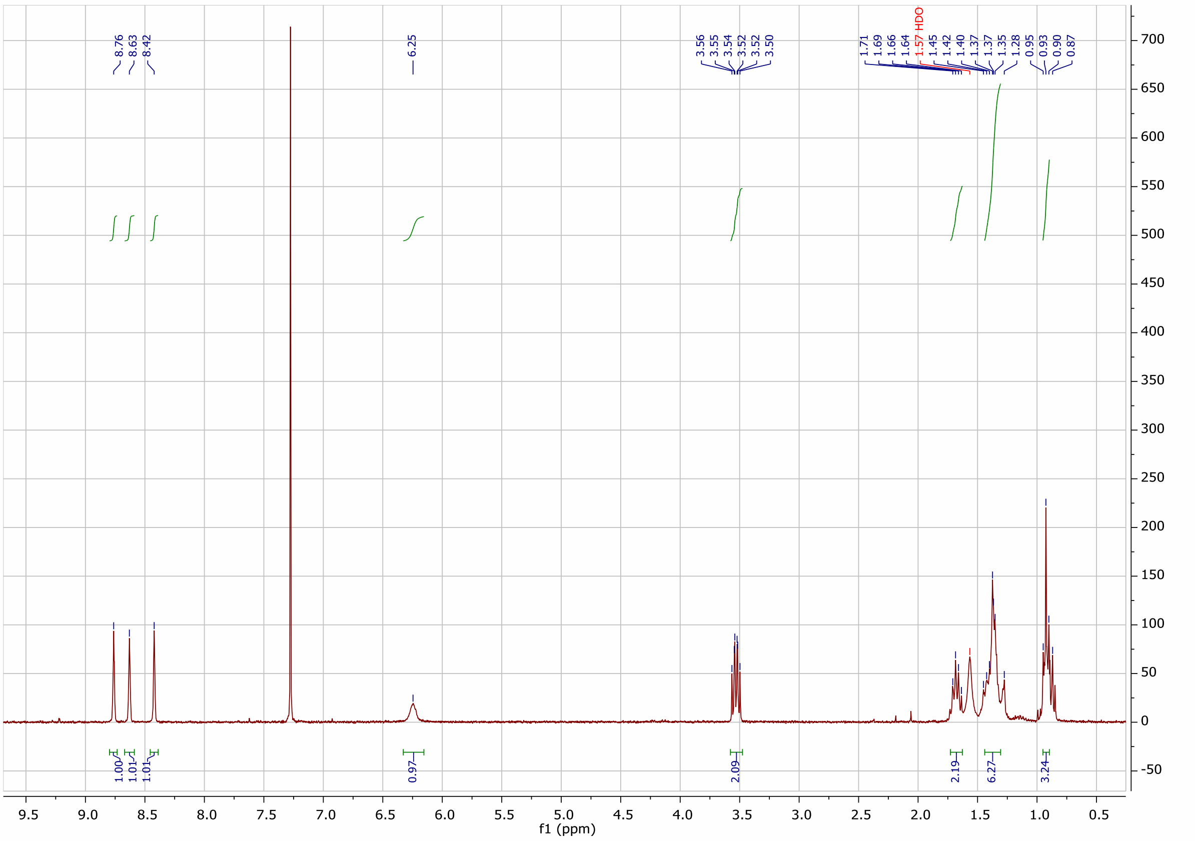


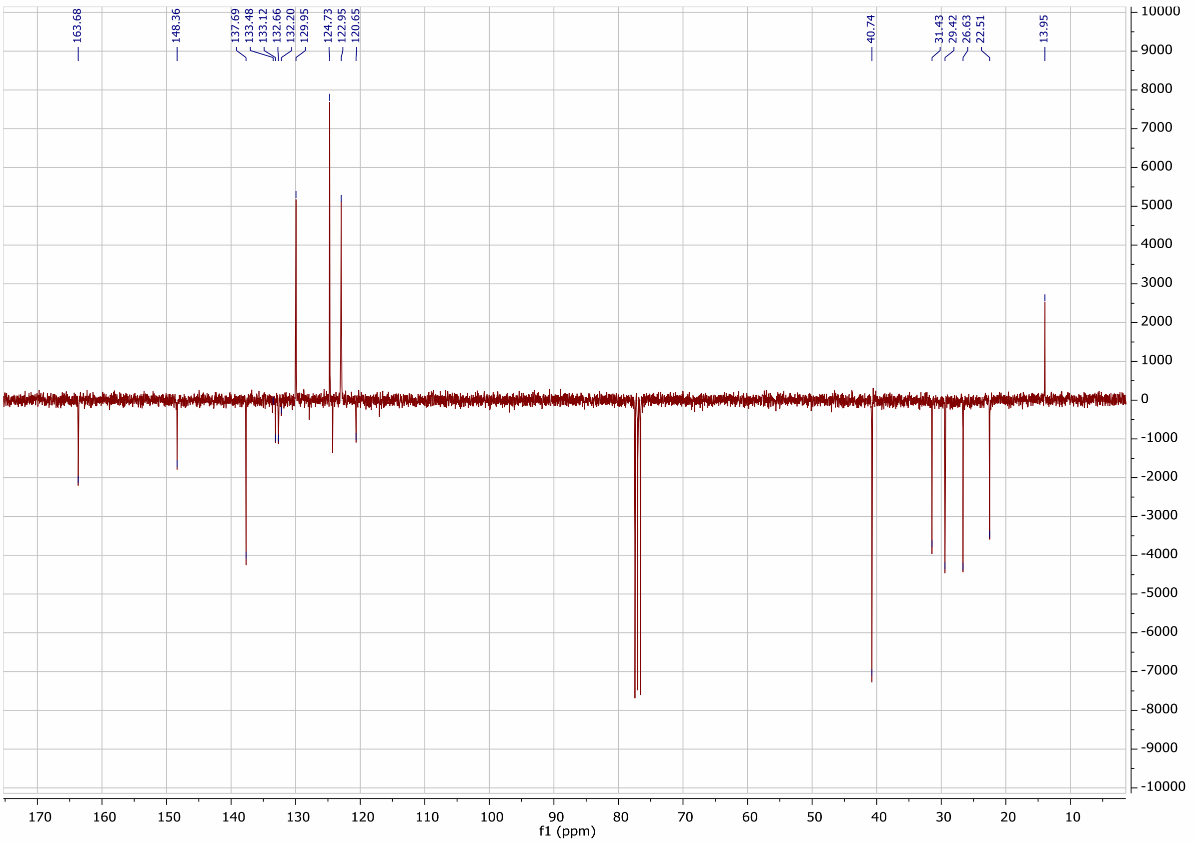

# *N*-octyl-3-nitro-5-(trifluoromethyl)benzamide **(18)** - $^{1}$H and $^{13}$C NMR spectra

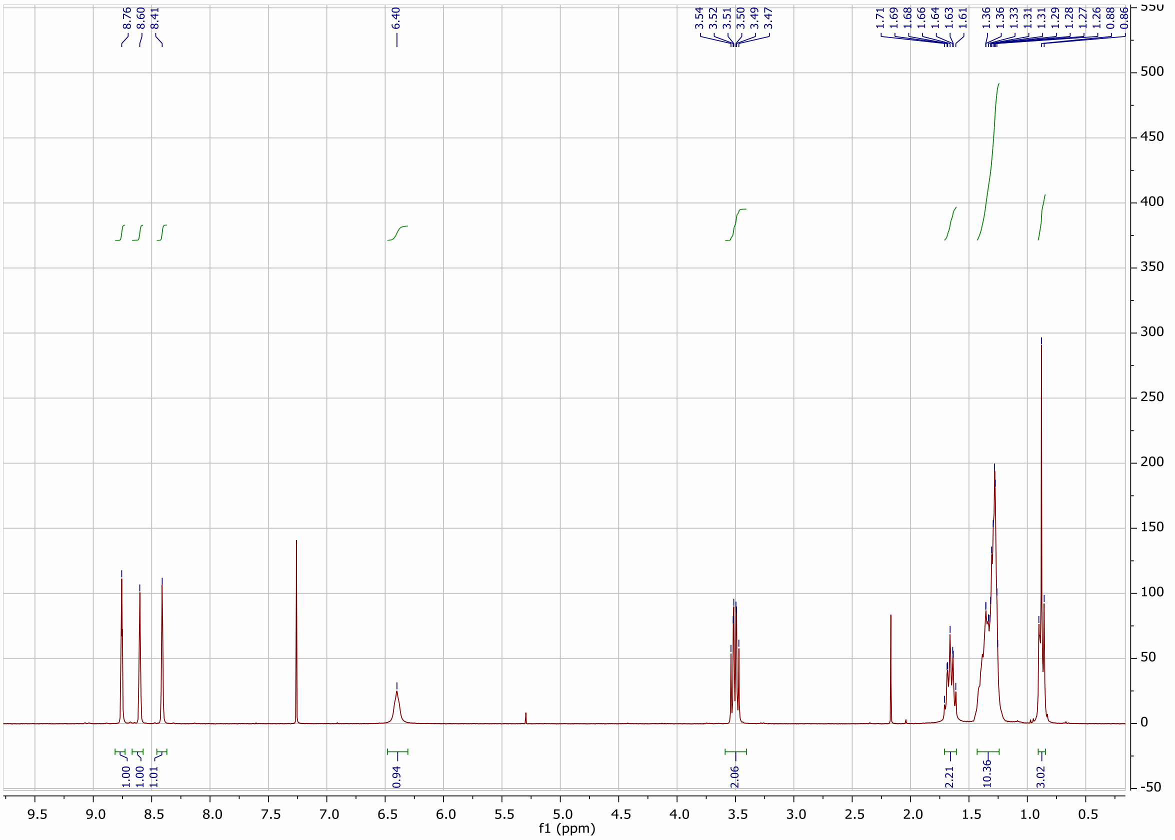


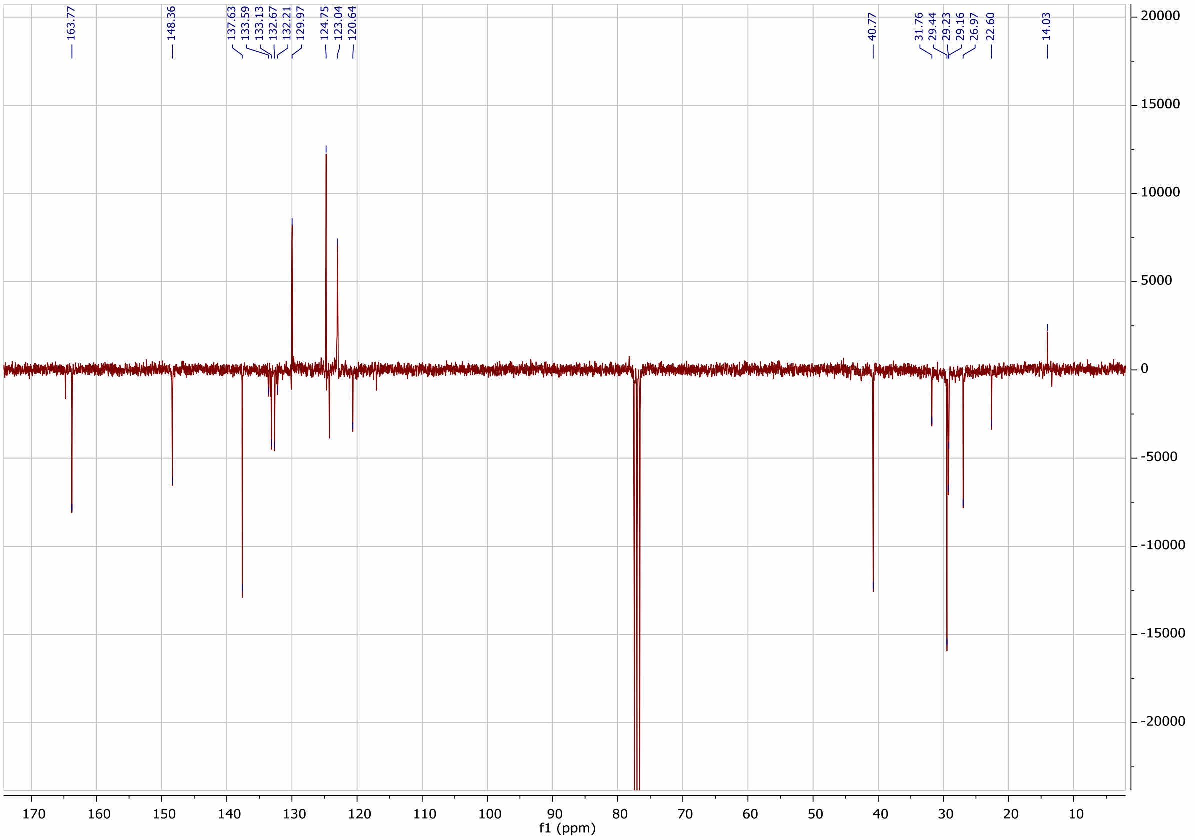

*N*-decyl-3-nitro-5-(trifluoromethyl)benzamide **(19)** - $^{1}$H and $^{13}$C NMR spectra

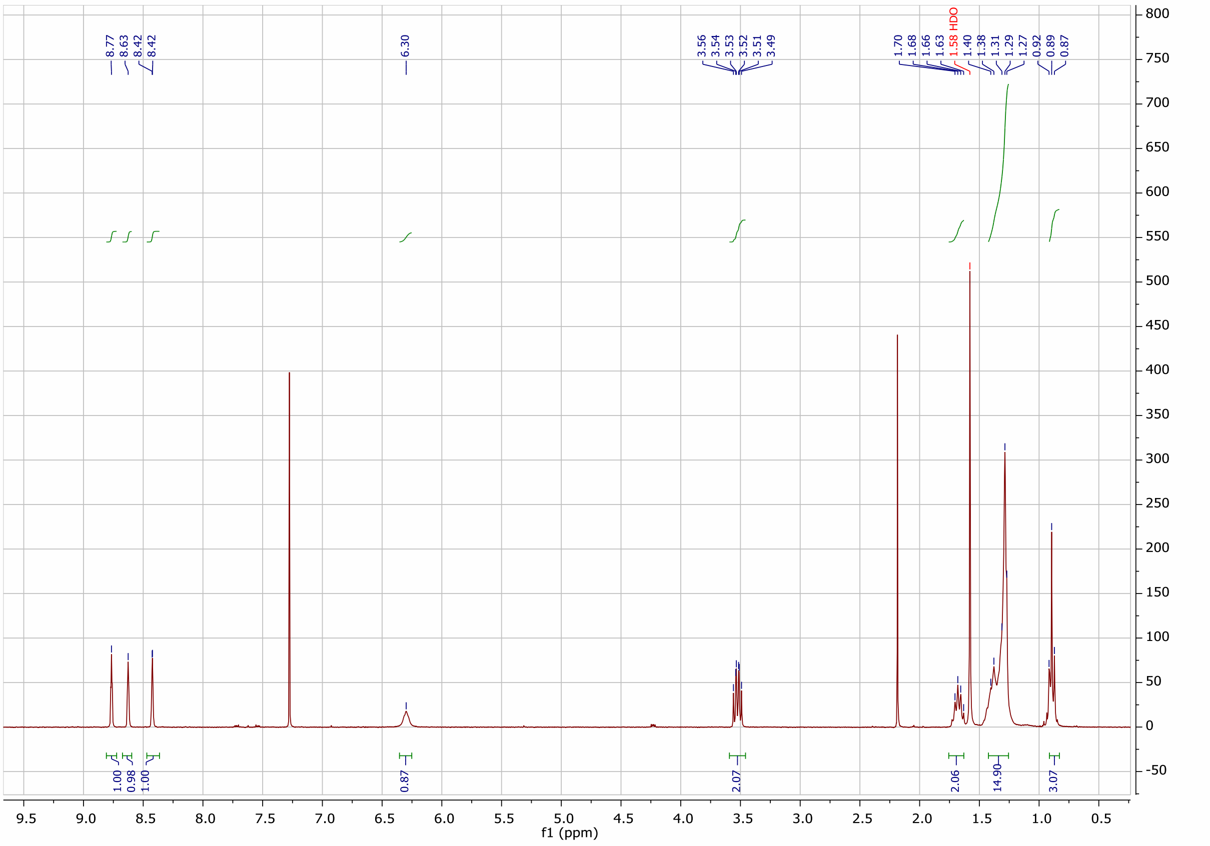


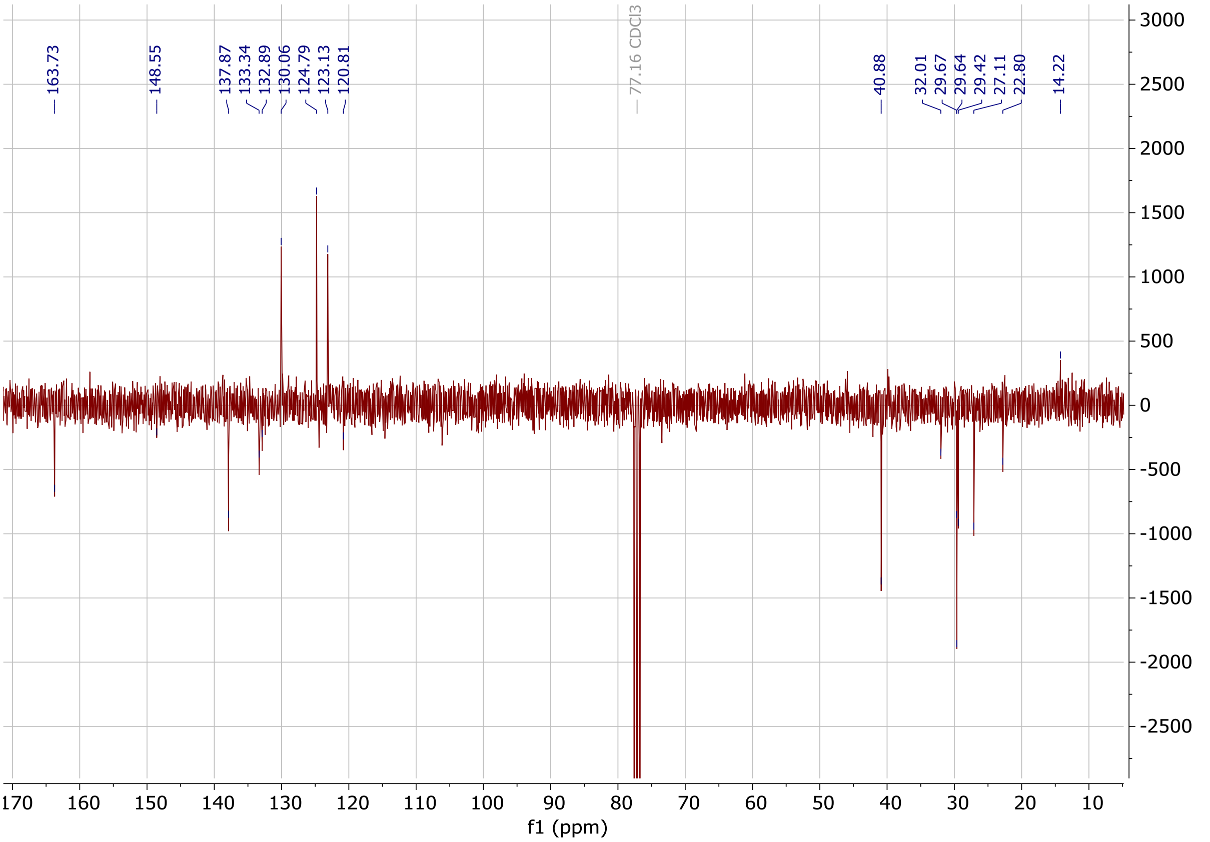

*N*-dodecyl-3-nitro-5-(trifluoromethyl)benzamide **(20)** - $^{1}$H and $^{13}$C NMR spectra

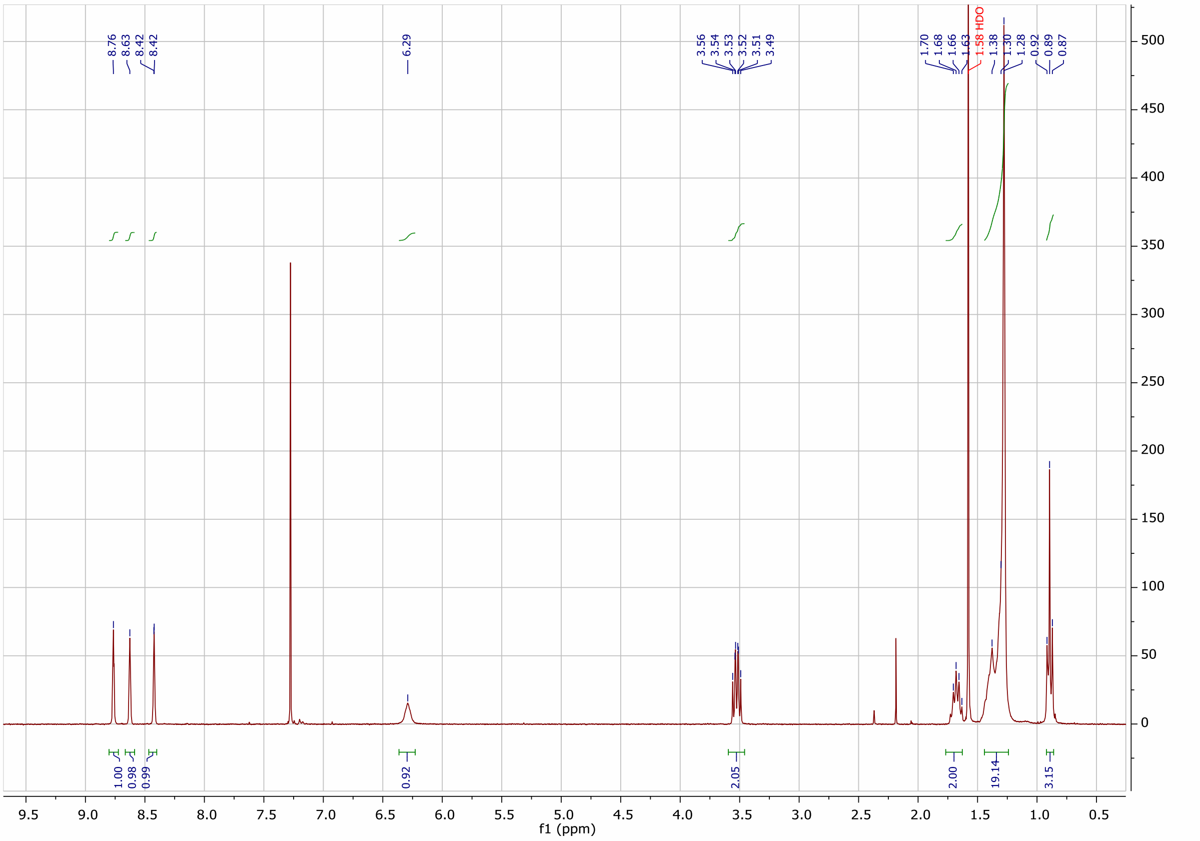


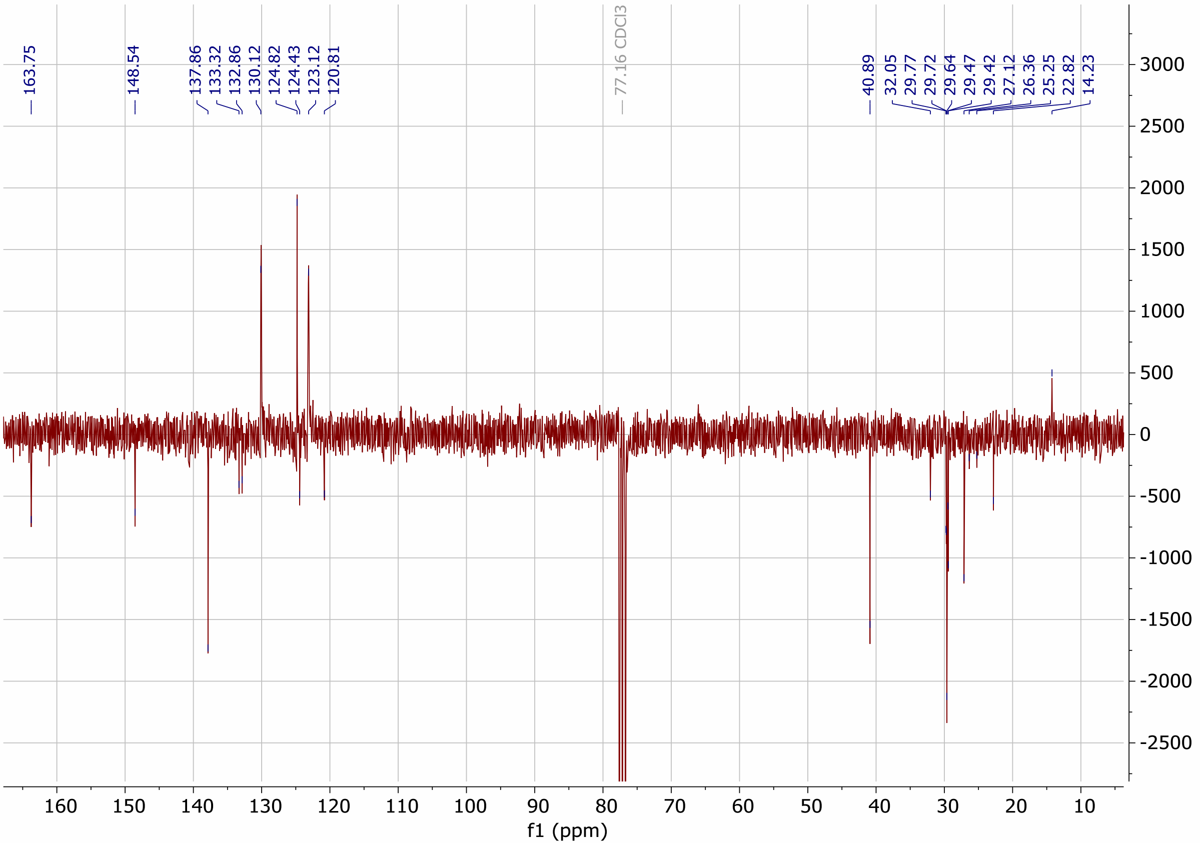

Supplement: Supplementary file 1 [file pharmaceuticals-17-00608-s001.zip › pharmaceuticals-2955761-supplementary.pdf]
